# Review on modeling theories of electrosensitive hydrogels for cartilage tissue engineering

**DOI:** 10.3389/fbioe.2025.1631725

**Published:** 2025-08-12

**Authors:** Abdul Razzaq Farooqi, Hermann Seitz, Ursula van Rienen

**Affiliations:** ^1^ Institute of General Electrical Engineering, University of Rostock, Rostock, Germany; ^2^ Department of Electronics Engineering, The Islamia University of Bahawalpur, Bahawalpur, Pakistan; ^3^ Faculty of Mechanical Engineering and Marine Technology, University of Rostock, Rostock, Germany; ^4^ Department Life, Light and Matter, University of Rostock, Rostock, Germany; ^5^ Department of Ageing of Individuals and Society, Interdisciplinary Faculty, University of Rostock, Rostock, Germany

**Keywords:** cartilage tissue engineering, electrical stimulation, electrosensitive hydrogels, scaffolds, transport theory, multiphasic theory, porous media theory, computational modeling

## Abstract

Electrosensitive hydrogels are smart biomaterials that swell, shrink, deform, and bend when an external electric field is applied. These hydrogels have enormous potential for the controlled therapeutic delivery of biochemical substances to the affected area, thus promoting tissue regeneration. Computational modeling and simulation approaches have provided researchers with cost-effective predictive models that can be used to optimize *in vitro* and *in vivo* experimental protocols. In this article, we present a review of the modeling theories that can be used for the modeling and numerical simulation of electrosensitive hydrogels immersed in a solution with an applied electric field for cartilage tissue engineering. Each theory presents tradeoffs for the numerical modeling of cartilage repair implants. The selection of an appropriate theory depends on the required accuracy, time-dependent application, and deformation behavior. Although most simulations are limited to one-dimensional cases, multidimensional simulations are crucial. By reviewing the modeling theories of electrosensitive hydrogels, this article aims to inspire researchers to model the electrical stimulation of electrosensitive hydrogels for various applications, including cartilage tissue engineering.

## 1 Introduction

Articular cartilage is a specialized connective tissue devoid of blood vessels, nerves, and lymphatics (Ngo and Knothe Tate, 2020). Chondrocytes are the primary cells in the extracellular network of articular cartilage and are responsible for repair and maintenance. An essential feature of articular cartilage is an extremely low cell count (Bhosale and Richardson, 2008). The primary function of articular cartilage is to facilitate smooth articulation in the diarthrodial joints of the body (Xu et al., 2022). Cartilage tissue is constantly degraded owing to injury, immobility, or aging, eventually leading to osteoarthritis (Van Gelder et al., 2023). Unlike other connective tissues, articular cartilage has very little cell growth; therefore, it does not heal easily when damaged (Bhosale and Richardson, 2008).

The electrochemical properties of articular cartilage originate from the flow of electrolytes relative to the fixed negative charges attached to the proteoglycans (Jackson and Gu, 2009). The resulting electromechanical events in the tissue can be distinguished by diffusion potential, streaming potential, and the Donnan osmotic swelling pressure (Buschmann and Grodzinsky, 1995; Lai et al., 2000). Thus, the charged nature of the tissue is responsible for its electrokinetic properties. Upon applying a mechanical load, electric potentials are generated owing to the movement of electrolytes across the proteoglycans. Alternatively, the application of an external electric field generates stress in the cartilage tissue (Frank and Grodzinsky, 1987a; b; Liu et al., 2025). Consequently, it was hypothesized that external electric fields similar to those generated in native articular cartilage can positively affect the synthesis of necessary extracellular matrix components (Brighton et al., 2006; Culma et al., 2025).

Polyelectrolyte hydrogels can be used as substitute materials to restore and regenerate articular cartilage (Romischke et al., 2022). Hydrogels are three-dimensional (3D) polymers that can absorb large quantities of water or biological fluids without being soluble in appropriate physiological environments (Lei et al., 2022; Kaith et al., 2021). They are extensively used in drug delivery (Qureshi et al., 2019), tissue engineering (El-Husseiny et al., 2022), sensors/biosensors (Hu et al., 2019), actuators (Hu et al., 2019), and cell-based therapies (Mohamed et al., 2019). They are also being utilized for novel biomedical applications, such as alleviating postoperative pancreatic fistulas, which are distinguished by the leakage of digestive enzymes (He et al., 2024), treating diabetic wounds using cross-linked multifunctional hydrogels (Shi et al., 2023), and promoting Achilles tendon repair while preventing adhesion using injectable lubricative hydrogels (Cheng et al., 2025). Moreover, hydrogels are widely used in the development of multifunctional personal protective equipment (Zhang et al., 2024) and the preparation of efficient and sustainable flame-retardant materials (Zuo et al., 2024). Hydrogels in general, and electrosensitive hydrogels in particular, are widely used to repair and regenerate the articular hyaline cartilage (Ni et al., 2023; Hashemi-Afzal et al., 2025). Stimuli-responsive hydrogels respond to external environmental stimuli and can be used in various applications (Roy et al., 2022). Several stimulation types exist for hydrogels, which can be classified into physical (stress, temperature, light, ultrasound, and electric and magnetic fields), chemical (ionic strength, pH), and biological (enzyme, glucose) stimuli-responsive hydrogels (Qureshi et al., 2019; El-Husseiny et al., 2022).

The prominent representatives of electrosensitive hydrogels are conjugated polymer-based compounds, such as polyaniline (PANi), polypyrrole (PPy), polythiophene, poly (3,4-ethylenedioxythiophene) (PEDOT), carbon-based materials, e.g., derivatives of graphene and carbon nanotubes (CNTs), hydrogels having gold and silver nanoparticles, and bio-ionic liquids (Carayon et al., 2020; Kanaan and Piedade, 2022; Walker et al., 2019). Electroresponsive hydrogels are gaining popularity because they have the distinct advantage of the precise and software-programmable control of electrical parameters (Erol et al., 2019). These hydrogels engineered with electrosensitive characteristics can revive the intrinsic electrochemical communication among cells, thereby augmenting tissue biofunction, which is hindered by injury (Lavrador et al., 2021; Gaspar et al., 2020). Such hydrogels are widely used in various biomedical applications (Ali et al., 2019; Rogers et al., 2020; Zou et al., 2022), including articular cartilage regeneration (Ni et al., 2023; Miguel et al., 2022; Zhou et al., 2023). Polymer-based electroactive hydrogels, such as PANi, PPy, and PEDOT, have been used for chondrocyte proliferation and differentiation (Hosseini et al., 2019; Uzieliene et al., 2023; Liu et al., 2023). Similarly, electrosensitive hydrogels containing gold and silver nanoparticles have been used for cartilage tissue engineering (Bai et al., 2023; He et al., 2015). Graphene, its derivatives (graphene oxide and reduced graphene oxide), and CNTs are widely used for the repair and regeneration of articular cartilage and osteochondral defects (Liu et al., 2021; Amiryaghoubi et al., 2023).

**FIGURE 1:**
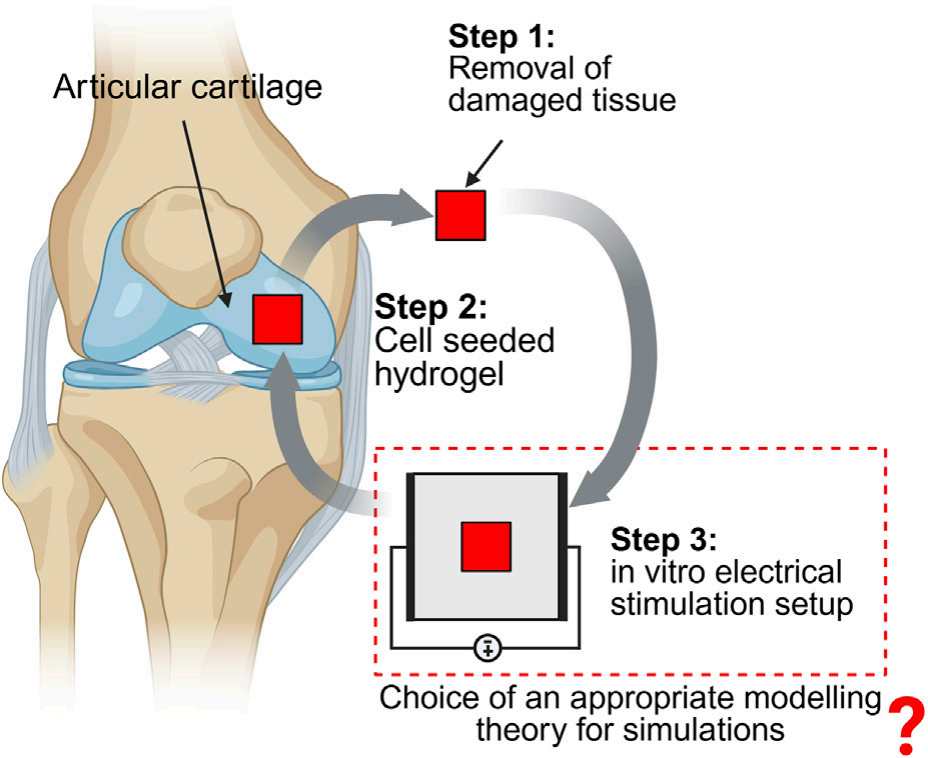
Different steps involved in articular cartilage tissue engineering using electrical stimulation (Created in Biorender).

Intrinsic electric fields are involved in various tissue functions including regeneration, disease expression, and progression (Zhao et al., 2020). Thus, extrinsic electric fields, similar to intrinsic electric fields, can be applied to cell-seeded scaffolds *in vitro* to develop therapeutic interventions (Ryan et al., 2021). These hydrogel scaffolds can then be implanted at the defect site, as illustrated in Figure 1. In this review, we focus on the *in vitro* electrical stimulation part of the tissue engineering approach and review the different theories that can be utilized for its modeling and simulation, as depicted in Figure 1. Such *in vitro* direct electrical stimulation setups have been used in various cartilage studies (MacGinitie et al., 1994; Chao et al., 2000; Hiemer et al., 2018). Similarly, capacitively coupled *in vitro* cartilage stimulation setups have been widely investigated (Brighton et al., 2008; Krueger et al., 2021; Esfandiari et al., 2014; Vaca-González et al., 2020). For a detailed description of electrical stimulation studies involving the cartilage tissue, interested readers can refer to our recent review (Zimmermann et al., 2024) or elsewhere (Vaca-González et al., 2019).

The swelling mechanism of hydrogels owing to stimulation can be explained by Flory’s osmotic pressure theory (Shiga and Kurauchi, 1990; Yang et al., 2000). As the hydrogel was immersed in the solution, the ionic species diffused between the hydrogel and the solution. Fixed charges are also present within the hydrogel. Thus, varying ionic concentrations were created between the hydrogel and the surrounding buffered solution. These variations in concentration result in osmotic pressure, which determines the swelling behavior of the hydrogel. The swelling and shrinking of the hydrogel caused a redistribution of the ionic species within the hydrogel until an equilibrium was established.

Several theories have been proposed to mathematically estimate the behavior of hydrogels placed in a solution under external electrical stimulation. These theories can be classified as the multiphasic or multi-effect-coupling electric-stimulus (MECe) theory (Li et al., 2004c; Chen et al., 2005), transport theory (Bassetti et al., 2005; Suthar et al., 2007; Wallmersperger and Ballhause, 2008), and porous media theory (PMT) (Wallmersperger et al., 2013b; Ehlers, 2002). The multiphasic theory arises from the multiphase mixture model of hydrated soft tissues (Gu et al., 1998). Mixture models are further classified into biphasic (Mow et al., 1980), triphasic (Lai et al., 1991), quadphasic (Huyghe and Janssen, 1997), and multiphasic (Gu et al., 1998) theories. The multiphasic model provides a detailed description of the solvent phase, ionic concentration, and deformation of the polymer network.

**FIGURE 2:**
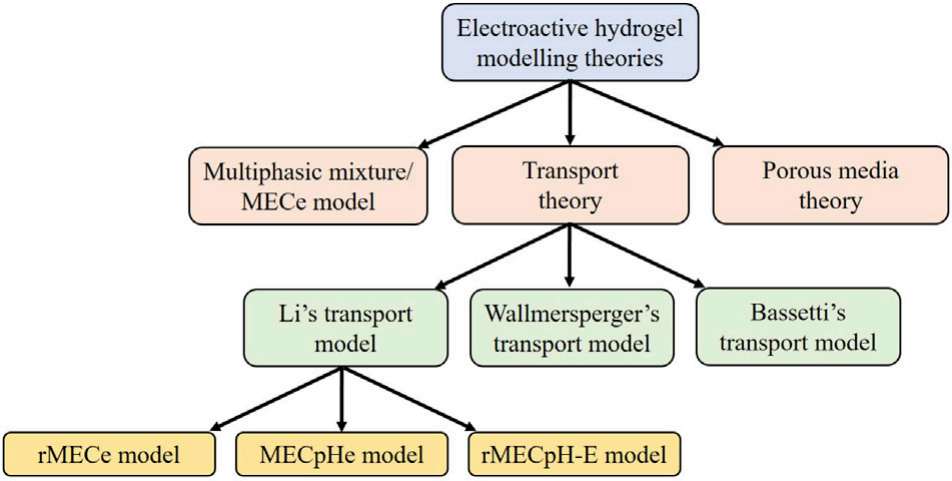
Modeling theories of the electrosensitive hydrogels. MECe is the multi-effect-coupling electric-stimulus model, rMECe is the refined multi-effect-coupling electric-stimulus model, MECpHe is the multi-effect-coupling pH-electric-stimuli model, and rMECpH-E represents the refined multi-effect-coupling pH-electric-stimuli model.

Three types of transport models exist in the literature, proposed by Li et al. (2004a), Wallmersperger (2003); Wallmersperger et al. (2004a), and Bassetti et al. (2005), and named Li’s, Wallmersperger’s, and Bassetti’s transport models, respectively. Different fluid or osmotic pressure equations lead to different transport models. In the following sections, we briefly describe the various theories used to describe the dynamics of electrosensitive hydrogels and compare them. All the mathematical models for the simulation of the electrosensitive hydrogels presented in this study are summarized in Figure 2.

We previously presented a numerical model for the simulation of electrosensitive hydrogels in the context of cartilage tissue engineering (Farooqi et al., 2019b; 2020). Such models have also been employed for the electrical stimulation of scleral tissue (Mehr and Hatami-Marbini, 2022; 2023; Hatami-Marbini and Mehr, 2022). Reviews of modeling theories for the electrical stimulation of hydrogels have been reported (Saunders et al., 2008; Wallmersperger and Leichsenring, 2016). However, to date, no review has provided a comprehensive and systematic overview of modeling approaches for the electrical stimulation of hydrogels, which reflects the current state of research. While previous reviews have laid important groundwork, they have not captured the significant theoretical and methodological advances made in recent years. This review fills this gap by critically analyzing the latest scientific contributions and integrating them with earlier findings. Thus, it offers the first consolidated and up-to-date reference in this rapidly evolving field, helping researchers navigate existing models and identify directions for future research.

In the following section, we review the modeling theories of electrosensitive hydrogels immersed in a solution and subjected to an external electric field. First, the constitutive equations of the involved chemo-electro-mechanical fields involved in each theory are introduced. Subsequently, the significant parameters and properties are described. The important features and limitations of these theories are summarized for readers interested in designing and optimizing experimental protocols for cartilage tissue engineering. Moreover, the experimental verification of the reported modeling theories and the type of hydrogel used for this purpose are discussed. Finally, conclusions are drawn, and possible directions for future research regarding the modeling theories of electrosensitive hydrogels for cartilage repair are highlighted.

## 2 Multiphasic mixture/multi-effect-coupling electric-stimulus (MECe) model

The MECe model includes the Poisson, Nernst–Planck, and continuum equations (including the continuity and momentum equations) for the deformation of hydrogels (Chen, 2004). The nonlinear MECe model is also known as a multiphasic mixture model. The Poisson equation corresponds to the electric potential and is coupled with the Nernst–Planck equations to calculate the ionic concentrations (Li et al., 2004c). The change in ionic concentration was then used to evaluate the hydrogel deformation using continuity and momentum equations (Saunders, 2013). The mathematical relationships of the MECe model, including the Poisson, Nernst–Planck, continuity, and momentum equations are discussed below.

### 2.1 Poisson equation

To determine the electric potential ψ , the Poisson equation is employed (Li et al., 2006),

$$\nabla^{2}\psi +\frac{F}{\varepsilon_{r}\varepsilon_{o}}\left(\overset{n}{\underset{k=1}{\sum }}z^{k}c^{k}+z^{f}c^{f}\right)=0$$ (1)

where $\varepsilon_{r}$ is the relative permittivity of the medium, $\varepsilon_{o}$ is the dielectric constant of vacuum, F is Faraday’s constant, $c^{k,f}$ are the ionic concentrations having valence $z^{k,f}$ , and $c^{f}$ and $c^{k}$ represent the fixed and mobile charge concentrations, respectively.

### 2.2 Nernst–Planck equation

The sum of the diffusion flux, electric transference, and convection-induced transfer of an ion k is called the total ionic flux ${\text{J}}^{k}$ , which is written as (Helfferich, 1962),

$${\text{J}}^{k}=-\left(D^{k}\nabla c^{k}+D^{k}c^{k}\nabla \text{ln}f^{k}+z^{k}\mu^{k}c^{k}\nabla \psi \right)+c^{k}\nu .$$ (2)

Here, $D^{k}$ and $\mu^{k}$ are the diffusivity and mobility of the ion *k*, respectively. $f^{k}$ is the activity coefficient for the diffusion flux, and ν is the area-averaged fluid velocity.

The continuity equation of the ionic flux for the entire domain is (Chen and Ma, 2006),

$$\frac{\partial c^{k}}{\partial t}+\nabla \cdot {\text{J}}^{k}=0.$$ (3)

In the absence of convection and chemical reactions, Equation 3 can be written as (Nernst, 1888; 1889; Planck, 1890),

$$\frac{\partial c^{k}}{\partial t}=D_{k}\nabla^{2}c^{k}+D_{k}\frac{z^{k}F}{RT}\nabla \cdot \left(c^{k}\nabla \psi \right)$$ (4)

where T is the absolute temperature and R is the gas constant. For the complete derivation of the Nernst–Planck equation, readers can refer to our previous publications (Farooqi et al., 2020; Farooqi, 2020) or elsewhere (Helfferich, 1962).

### 2.3 Continuity equation

According to the law of mass conservation, the solid, liquid, and ionic phases have velocities (Li et al., 2007a)

$$\frac{\partial \rho^{\alpha }}{\partial t}+\nabla \cdot \left(\rho^{\alpha }{\text{v}}^{\alpha }\right)=0$$

where ${\text{v}}^{\alpha }$ and $\rho^{\alpha }$ denote the velocity and local mass density of the component α , respectively.

From the triphasic theory (Lai et al., 1991), the fixed charge density is dependent upon the tissue deformation of tissue and the volume fraction of fluid $\phi^{w}$ (porosity). Thus,

$$\frac{\partial \left(\phi^{w}c^{f}\right)}{\partial t}+\nabla \cdot \left(\phi^{w}c^{f}{\text{v}}^{s}\right)=0$$

which is represented with the tissue deformation as

$$c^{f}=\frac{c_{o}^{f}}{1+\text{tr}\left(\text{E}\right)/\phi_{o}^{w}}$$ (5)

and the solidity of the tissue $\phi^{s}$ is

$$\phi^{s}=\frac{\phi_{o}^{s}}{1+\text{tr}\left(\text{E}\right)}$$

where $c_{o}^{f}$ is the initial fixed charge density. $\phi_{o}^{s}$ and $\phi_{o}^{w}$ are the volume fractions of the solid and fluid phases, respectively, in the reference configuration. Furthermore, **E** denotes the strain tensor of the initial geometry relevant to the saturated solution.

The magnitudes of $\phi^{k}$ are negligible compared with $\phi^{s}$ and $\phi^{w}$ ; hence, the saturation condition is simplified to

$$\phi^{s}+\phi^{w}=1,$$

and we have

$$\phi^{w}=1-\frac{\phi_{o}^{s}}{1+\text{tr}\left(\text{E}\right)}.$$ (6)

The saturation condition (9) for the reference configuration can be written as,

$$\phi_{o}^{s}+\phi_{o}^{w}=1.$$ (7)

Ignoring the inertial and body forces, the governing equations of the multiphasic theory can be written as

$$\sigma =-p\text{I}+\lambda_{s}\text{tr}\left(\text{E}\right)\text{I}+2\mu_{s}\text{E}$$ (8)

$$\mu^{w}=\mu_{o}^{w}+\frac{1}{\rho_{T}^{w}}\left(p-RT\overset{n}{\underset{k=1}{\sum }}\varphi^{k}c^{k}+B_{w}\text{tr}\left(\text{E}\right)\right)$$

$$\mu^{k}=\mu_{o}^{k}+\frac{RT}{M^{k}}\text{ln}\left(\gamma_{k}c^{k}\right)+z^{k}\frac{F\psi }{M^{k}}$$

where $\gamma_{k}$ and $M^{k}$ are the activity coefficient and the molar weight of the ion *k*, respectively. Furthermore, p is the fluid pressure, σ is the stress tensor, $\mu_{o}^{\alpha }\;\left(\alpha =w,k\right)$ are the chemical potentials of the phase α for the initial geometry, **I** is the identity tensor, $B_{w}$ is the coupling coefficient, and $\varphi^{k}$ is the osmotic coefficient of the ion k .

Thus, the continuity equation of the multiphasic theory for the fluid pressure p is,

$$\nabla \cdot \frac{\partial u^{s}}{\partial t}=\nabla \cdot [\frac{{\left(\phi^{w}\right)}^{2}}{f_{ws}}(\nabla p-RT\left(\varphi^{k}-1\right)\nabla \overset{n}{\underset{k=1}{\sum }}c^{k}+F\overset{n}{\underset{k=1}{\sum }}z^{k}c^{k}\nabla \psi +B_{w}\nabla \text{tr}\left(\text{E}\right))]$$ (9)

where $f_{ws}$ is the friction coefficient and $u^{s}$ is the solid phase displacement. Here $\phi^{k}=1$ and $B_{w}=0$ ; thus Equation 9 becomes

$$\nabla \cdot \frac{\partial u^{s}}{\partial t}=\nabla \cdot \left[\frac{{\left(\phi^{w}\right)}^{2}}{f_{ws}}\left(\nabla p+F\underset{k=+,-}{\sum }z^{k}c_{k}\nabla \psi \right)\right].$$ (10)

Only a few important equations relevant to the continuity equation were presented. The detailed derivation of the continuity equation can be found in our previous study (Farooqi et al., 2019a).

### 2.4 Momentum equation

The momentum equation for estimating the displacement when the deformations are small is written as (Chandrasekharaiah and Debnath, 1994; Tanaka et al., 1973)

$$\rho \frac{\partial^{2}\text{u}}{\partial t^{2}}+\zeta \frac{\partial \text{u}}{\partial t}=\nabla \cdot \sigma +\rho \text{b}$$ (11)

where ρ is the effective hydrogel density, u is the displacement vector, ζ is the viscous damping, and b represents the body force (Chandrasekharaiah and Debnath, 1994). The frictional effects can be ignored because the concentration of mobile ions is assumed to be very low, and Equation 11 becomes

$$\rho \frac{\partial^{2}\text{u}}{\partial t^{2}}=\nabla \cdot \sigma .$$ (12)

The redistribution of ions occurs at a much lower speed than that of hydrogel deformation. Thus, a quasi-static condition can be considered, and Equation 12 is reduced to

∇·σ=0.

**FIGURE 3:**
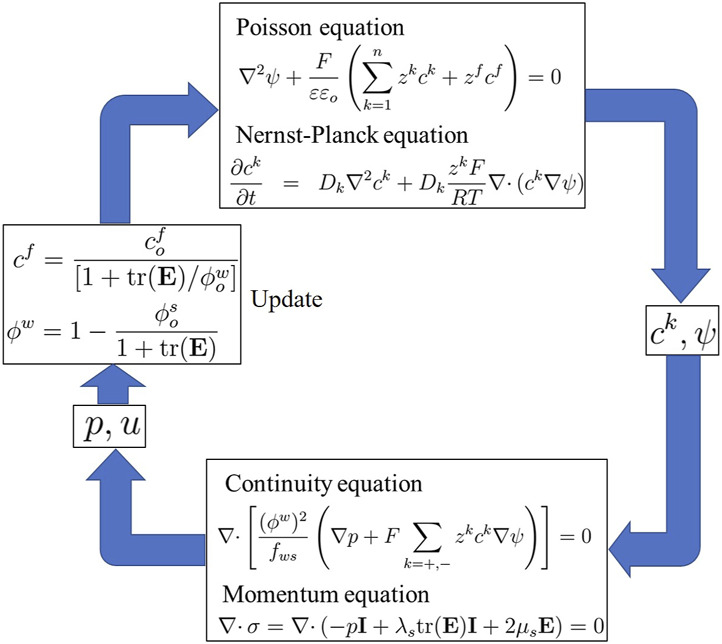
Simulation flow diagram of the multiphasic mixture/MECe model.

In addition, the electrostatic stress was neglected because of the small potential difference applied. Using Equation 8, the above equation becomes

$$\nabla \cdot \sigma =\nabla \cdot \left(-p\text{I}+\lambda_{s}\text{tr}\left(\text{E}\right)\text{I}+2\mu_{s}\text{E}\right)=0$$ (13)

where $\lambda_{s}$ and $\mu_{s}$ are the Lamé constants. Hence, Equations 1, 2, 9 and 13, along with Equations 5 and 6, collectively constitute the multiphasic mixture or MECe model (Li, 2009a). Figure 3 shows the complete simulation of this model.

## 3 Wallmersperger’s transport model

The transport model for the electrical stimulation of hydrogels proposed by Wallmersperger et al. (2004a), (2004c) consists of the Poisson–Nernst–Planck, osmotic pressure, and momentum equations. The Poisson, Nernst–Planck, and momentum equations have been expressed in detail in Equations 1, 4 and 13, respectively. The remaining osmotic pressure equation is as follows:

### 3.1 Osmotic pressure equation

In the Wallmersperger transport model, the fluid pressure in Equation 10 is modified by the osmotic pressure $p_{\mathrm{osm}}$ (Wallmersperger et al., 2004b; 2009). It depends on the concentration of ions and is evaluated using the following equation (Horkay et al., 2000):

$$p_{\mathrm{osm}}=RT\underset{k=+,-}{\sum }\left(c_{\mathrm{gel}}^{k}-c_{\mathrm{sol}}^{k}\right)$$ (14)

where $c_{\mathrm{gel}}^{k}$ and $c_{\mathrm{sol}}^{k}$ represent the concentrations of *k* ions inside the hydrogel and in the solution immediately outside the hydrogel, respectively. Therefore, the equation of linear elasticity that governs the deformation of the hydrogel in the equilibrium state is written using Equation 13 as (Biot, 1956)

$$\nabla \cdot \sigma =\nabla \cdot \left(-p_{\mathrm{osm}}\text{I}+\lambda_{s}\text{tr}\left(\text{E}\right)\text{I}+2\mu_{s}\text{E}\right)=0.$$

**FIGURE 4:**
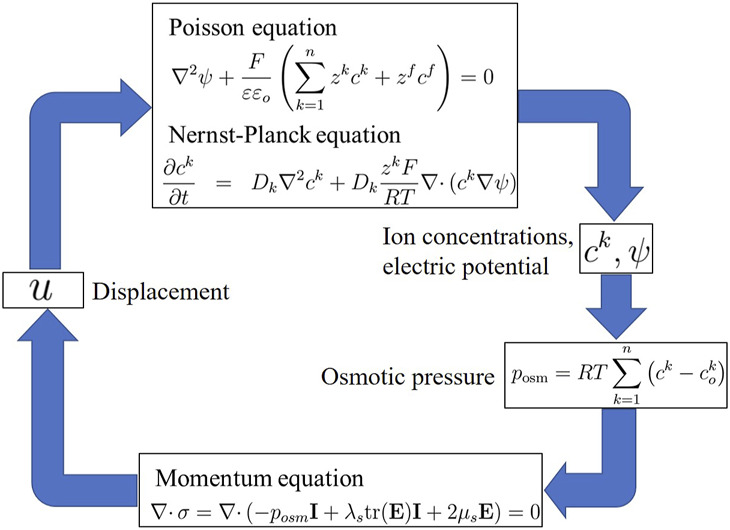
Simulation flow diagram of Wallmersperger’s transport model.

Thus, Equations 1, 4 and 13, and 14 collectively constitute the transport model proposed by Wallmersperger, as shown in the simulation flow diagram in Figure 4.

## 4 Li’s transport model

Several variations of the transport model proposed by Yew (2006), Luo (2008) exist for the electrical excitation of hydrogels in a solution. These variations of the transport model are classified into the refined multi-effect-coupling electric-stimulus model (rMECe) (Lam et al., 2006), the multi-effect-coupling pH-electric-stimuli model (MECpHe) (Luo et al., 2007b), and the refined multi-effect-coupling pH-electric-stimuli model (rMECpH-E) (Li et al., 2004b; 2005) to include nonlinear deformation under the simultaneous variation of pH and electric field (Yew et al., 2007). All these transport models use the Poisson equation to evaluate the electric potential, the Nernst–Planck equation for ionic concentration profiles, and the equation of motion to estimate the deformation of the hydrogels. These models differ in how the fluid/osmotic pressure is coupled from the Poisson–Nernst–Planck equations to the equation of motion. A mathematical description of each Li transport model is provided below.

### 4.1 Refined multi-effect-coupling electric-stimulus (rMECe) model

The multi-effect-coupling pH-stimulus model (MECpH) (Li et al., 2004b; 2005) describes hydrogel swelling behavior, which is sensitive to pH variation. The rMECe is a refined version of the MECpH model that can be extended to electroresponsive hydrogels (Luo et al., 2007a; Li et al., 2007d). The rMECe model has a reformulated fixed-charge density to accommodate an external electric field. Additionally, the refined model could incorporate large deformations of the hydrogels at a high applied voltage.

From Equations 6 and 7, the volume fraction of the fluid phase is

$$\phi^{w}=1-\frac{1-\phi_{o}^{w}}{1+\text{tr}\left(\text{E}\right)}=\frac{\text{tr}\left(\text{E}\right)+\phi_{o}^{w}}{1+\text{tr}\left(\text{E}\right)}.$$ (15)

Combining the triphasic theory (Lai et al., 1991) and Equation 15 yields

$$c^{f}=\frac{\phi_{o}^{w}c_{o}^{f}}{\phi_{o}^{w}+\text{tr}\left(\text{E}\right)}=\frac{c_{o}^{f}}{\left[1+\text{tr}\left(\text{E}\right)/\phi_{o}^{w}\right]}$$ (16)

where $c_{o}^{f}$ is the initial concentration of fixed charges of the hydrogel.

To include the effects of large deformations in the rMECe model, the nonlinear governing equations were described using the total Lagrangian formulation as (Li et al., 2007c)

$$\nabla \cdot \text{P}=\nabla \cdot \left({\text{SF}}^{T}\right)=0\;\text{in}\;\Omega ,$$ (17)

$$\text{u}=\text{G}\;\text{in}\;\Gamma_{g},$$ (18)

$$\text{P}\cdot \text{N}=\text{H}\;\text{in}\;\Gamma_{h},$$ (19)

where P and S represent the first and second Piola–Kirchhoff stress tensors, respectively. Similarly, G and H are the specified displacement and surface traction vectors on boundaries $\Gamma_{g}$ and $\Gamma_{h}$ , respectively. F is the deformation gradient tensor and N is the unit outward normal vector. The displacement vector u denotes the displacement from the initial state X to the deformed state x , such that x=X+u . The first Piola–Kirchhoff stress tensor is asymmetrical and immeasurable; therefore, the second Piola–Kirchhoff stress tensor is required because it is symmetric and can be utilized as a stress measure given as

$$\text{S}=\text{CE}-p_{\mathrm{osm}}\text{I}$$ (20)

where C is the material tensor.

Substituting Equation 20 into Equation 17, the hydrogel governing equation for large deformation becomes (Luo et al., 2008a)

$$\nabla \cdot \left[\left(\text{CE}-p_{\mathrm{osm}}\text{I}\right){\text{F}}^{T}\right]=0.$$ (21)

**FIGURE 5:**
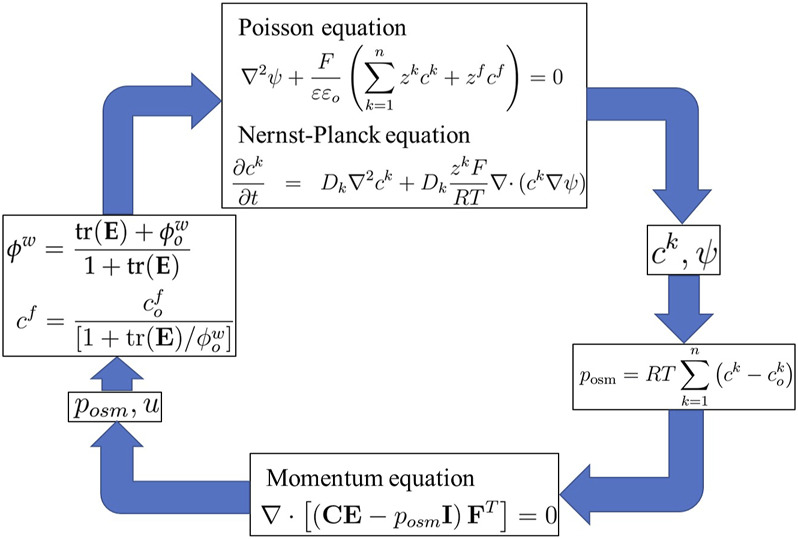
Simulation flow diagram of Li’s transport/rMECe model.

Owing to the low applied voltage, the hydrogels exhibited small deformations. The linear theory is sufficient and can be evaluated using Equation 13. Thus, Equations 1, 2, 15, 16 and 21 collectively constitute Li’s transport model or the rMECe model, as shown in the simulation flow diagram in Figure 5.

### 4.2 Multi-effect-coupling pH-electric-stimuli (MECpHe) model

The MECpHe model for simulating hydrogel swelling and deformation with the simultaneous effects of the applied electric field and solution pH is presented herein (Li et al., 2007b; Luo et al., 2008b). The hydrogel is considered to be a triphasic mixture of an ionic species, a solid matrix, and an interstitial fluid.

When an electric field is applied, mobile ions move to the opposite electrode, creating a gradient of ionic concentrations. The hydrogel structure has a fixed negative charge. An ionic concentration difference is created at the interface between the hydrogel and the buffer solution, resulting in osmotic pressure. The diffusion of cations in the hydrogel dominates that of anions. At the interface, the increase in osmotic pressure was greater near the anode than near the cathode, and the hydrogel swelled more at the anode than at the cathode.

The Langmuir absorption theory (Grimshaw et al., 1990) was used to represent the fixed charge density as

$$c^{f}=\frac{1}{1+H}\frac{c_{s}^{f}K}{K+c^{H}}$$ (22)

where H is the hydrogel hydration, defined as $H=V^{w}/V^{s}$ ( $V^{w}$ and $V^{s}$ represent the volumes of the interstitial fluid phase and dry hydrogel, respectively). K represents the dissociation constant of the fixed charges of the hydrogels, $c_{s}^{f}$ is the total concentration in dry conditions, and $c^{H}$ is the hydrogen ion ( $H^{+}$ ) concentration inside the hydrogel.

Using the relations between the volume fractions and hydration, we obtain

$$1+H=\frac{V^{s}+V^{w}}{V^{s}}=\frac{V}{V^{s}}=\frac{1}{\phi^{s}}=\frac{1}{1-\phi^{w}}$$ (23)

The fluid- and solid-phase volume fractions derived from Equation 23 are

$$\phi^{w}=\frac{H}{1+H}$$ (24)

$$\phi^{s}=\frac{1}{1+H}.$$

The relation of fluid and solid volume fractions, ignoring the ionic volume fraction $\phi^{k}$ , is

$$\phi^{w}=1-\frac{V^{s}}{V}=1-\frac{V^{s}}{V_{o}}\frac{V^{o}}{V}=1-\phi_{o}^{s}J$$ (25)

where $J\left(dV_{o}/dV\right)$ is the volume ratio of the apparent solid phase, described using Green’s strain tensor E as follows (Hon et al., 1999):

$$J^{-1}=\sqrt{1+2F_{1}\left(\text{E}\right)+4F_{2}\left(\text{E}\right)+8F_{3}\left(\text{E}\right)}$$

where $F_{1}\left(\text{E}\right)=\text{tr}\left(\text{E}\right)$ , $F_{2}\left(\text{E}\right)$ , and $F_{3}\left(\text{E}\right)$ represent the first, second, and third invariants^1^ of the tensor E , respectively.

Using Equations 24 and 25,

$$\phi^{w}=1-\phi_{o}^{s}J.$$ (26)

Rearranging Equation 26, the local hydration of the hydrogel is

$$H=\frac{1-\phi_{o}^{s}J}{\phi_{o}^{s}J}.$$ (27)

Substituting Equations 26 and 27 into Equation 22, the fixed charge density can be rewritten as

$$c_{f}=\frac{c_{s}^{f}K\phi_{o}^{s}}{\left(K+c^{H}\right)\sqrt{1+2F_{1}\left(\text{E}\right)+4F_{2}\left(\text{E}\right)+8F_{3}\left(\text{E}\right)}}.$$ (28)

**FIGURE 6:**
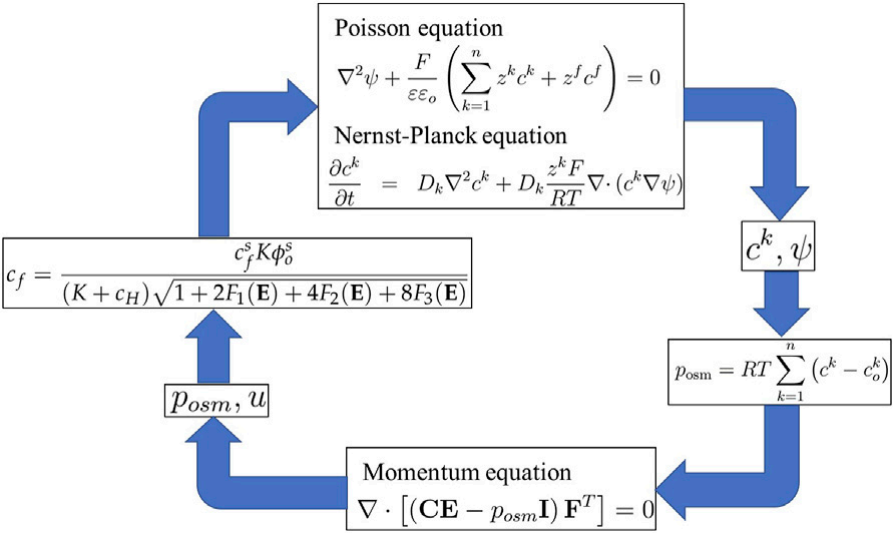
Simulation flow diagram of Li’s transport/MECpHe model.

The current MECpHe model is based on the finite elastic deformation theory instead of the small deformation theory to establish a mechanics equation. If pH-electric-sensitive hydrogels experience large deformations with the application of a high electric field, the linear elastic theory cannot provide reasonably accurate results (Luo et al., 2009). This is because the deviation between the initial and deformed states can no longer be ignored, as is the case for the linear elastic behavior. Consequently, for the case of nonlinear large deformation, mechanical equations based on the total Lagrangian description are used, as described in Equations 17–21 (Li et al., 2009; Luo et al., 2010). Thus, Equations 1, 2, 14, 21 and 28 collectively constitute Li’s transport/MECpHe model, as depicted in the simulation flow diagram in Figure 6.

### 4.3 Refined multi-effect-coupling pH-electric-stimuli (rMECpH-E) model

rMECpH-E is an improved version of the MECpH model that includes a finite/nonlinear deformation formulation in the equilibrium mechanical equation (Yew et al., 2007; Li et al., 2007e). An equation was developed based on the Langmuir absorption isotherm that describes the relationship between fixed charges and diffusing hydrogen ions (Grimshaw, 1989). The fixed charge concentration is given by

$$c^{f}=\frac{c_{o}^{f}K}{H\left(K+c^{H}\right)}.$$ (29)

The initial and final configuration differences cannot be ignored if the elastic body undergoes large deformation. Green–Lagrangian strain and second Piola–Kirchhoff stress tensors were incorporated for this type of analysis. The Lagrangian formulation provides the momentum equation for a large deformation as follows:

$$\nabla_{X}\cdot \text{P}+\text{b}=\rho \ddot{u}$$ (30)

where $\nabla_{X}$ represents the material derivative, and $\rho \ddot{u}$ is the inertial force. The first Piola–Kirchhoff stress tensor indicates the expanding and elastic retarding stresses as follows:

$$\text{P}=-J{\text{F}}^{-1}p_{\mathrm{osm}}\text{I}+\text{FS}$$ (31)

where J represents the determinant of deformation gradient F

J=detF.

The deformation gradient F is expressed as

$$\text{F}=F_{ij}=\frac{\partial x_{i}^{\text{Deformed}-\text{configuration}}}{\partial x_{i}^{\text{Initial}-\text{configuration}}}=\delta_{ij}+\frac{\partial u_{i}}{\partial X_{j}}=\text{I}+\nabla_{X}\text{u}.$$

The second Piola–Kirchhoff stress tensor S is expressed as

S=C:E (32)

where the symbol “:” represents the second-order inner product of two tensors (Zohdi, 2017). The Green–Lagrange strain E in Equation 32 is

$$\text{E}=\frac{1}{2}\left({\text{F}}^{T}\text{F}-\text{I}\right).$$

Similarly, for an elastic isotropic substance, the material tensor in Equation 32 can be expressed as

$$\text{C}=\left[\begin{array}{cccccc} \lambda_{s}+2\mu_{s} & \lambda_{s} & \lambda_{s} & 0 & 0 & 0\\ \lambda_{s} & \lambda_{s}+2\mu_{s} & \lambda_{s} & 0 & 0 & 0\\ \lambda_{s} & \lambda_{s} & \lambda_{s}+2\mu_{s} & 0 & 0 & 0\\ 0 & 0 & 0 & 0 & 0 & 0\\ 0 & 0 & 0 & 0 & 0 & 0\\ 0 & 0 & 0 & 0 & 0 & 0 \end{array}\right].$$

Substituting Equation 31 into Equation 30, the mechanical deformation equation for the rMECpH-E model becomes

$$\nabla_{X}\cdot \left(-J{\text{F}}^{-1}p_{\mathrm{osm}}\text{I}+\text{FS}\right)+\text{b}=\rho \ddot{u}.$$ (33)

If the inertial and body forces are ignored, Equation 33 can be simplified as

$$\nabla_{X}\cdot \left(-J{\text{F}}^{-1}p_{\mathrm{osm}}\text{I}+\text{FS}\right)=0\;\text{in}\;\Omega .$$ (34)

**FIGURE 7:**
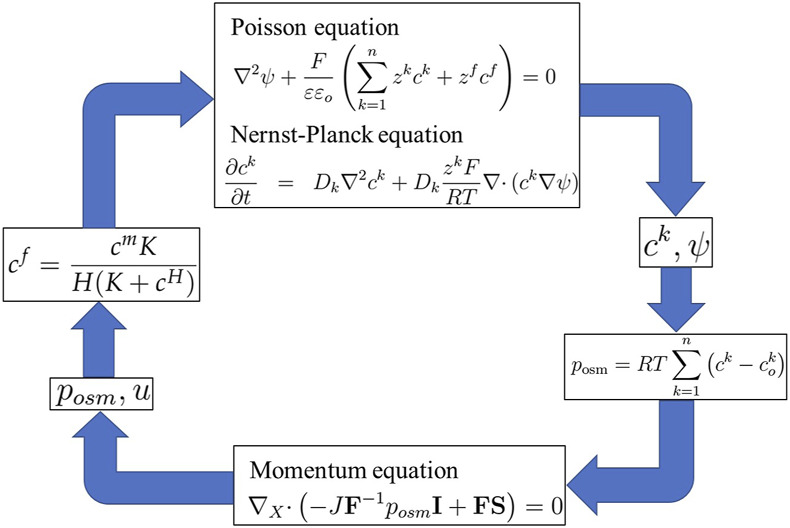
Simulation flow diagram of Li’s transport/rMECpH-E model.

The relevant boundary conditions are prescribed in Equations 18 and 19 (Ng et al., 2007). Hence, Equations 1, 2, 15, 29 and 34 collectively constitute Li’s transport model or the rMECpH-E model (Ng et al., 2010), as is evident from the simulation flow diagram in Figure 7.

## 5 Bassetti’s transport model

This model relies on the work of De et al. (2002), De and Aluru (2004), which was initially utilized for modeling pH-stimulated hydrogels, and later extended by Bassetti et al. (2005) for electrically stimulated hydrogels. Similar to other transport models, it also starts with the Poisson equation, described in Equation 1, for the external electric field and its interaction with mobile and fixed ions. The hydrogel has an effective dielectric constant $\varepsilon_{r}$ , which can be calculated by the relation (Nemat-Nasser and Li, 2000; Nemat-Nasser, 2002)

$$\varepsilon_{r}=\frac{\varepsilon_{p}+\varepsilon_{w}-\phi^{w}\left(\varepsilon_{p}-\varepsilon_{w}\right)}{\varepsilon_{p}+\varepsilon_{w}+\phi^{w}\left(\varepsilon_{p}-\varepsilon_{w}\right)}\varepsilon_{p}$$

where $\varepsilon_{p}$ and $\varepsilon_{w}$ represent the dielectric constants of the polymer and fluid, respectively. The fixed charge concentration is represented by Equation 29, and the Nernst–Planck Equation 2 was utilized for the flux of ionic species while considering the porosity of the hydrogel.

The porosity of a hydrogel is related to its hydration state, as shown in Equation 24. The Nernst–Planck Equation 2 is written specifically for hydrogen ions, assuming that neither chemical reactions nor convection occur (Saunders et al., 2008; Nussbaum, 1986)

$${\text{J}}^{H}=\phi^{w}\left(-D^{k}\nabla c^{k}-z^{k}\mu^{k}c^{k}\nabla \psi \right)+c^{k}\nu .$$ (35)

One way that this model differs from other transport models is how hydrogen ions ( $H^{+}$ ) are considered. Some hydrogen ions were attached to the fixed charges of the hydrogel. Another necessary modification of $H^{+}$ ions is to consider the number of ions generated from water at the anode. The continuity equation for hydrogen ions is as follows:

$$\frac{\partial }{\partial t}\left(Hc^{H}+Hc_{b}^{H}+Hc^{HB}\right)=\nabla \cdot \left({\text{J}}^{H}+{\text{J}}^{HB}\right)$$ (36)

where $c^{HB}$ is the concentration of hydrogen ions attached to the solution inside the hydrogel, and ${\text{J}}^{HB}$ is the flux of these ions. $c_{b}^{H}$ is the concentration of hydrogen ions attached to the charged groups on the polymer chain, and is expressed as

$$c_{b}^{H}=\frac{c^{\mathrm{tot}}c^{H}}{K+c^{H}}$$ (37)

where $c^{\mathrm{tot}}$ is the total ionic concentration of the buffer inside the hydrogel, which is the sum of $c^{HB}$ and the mobile buffer ion concentration, $c^{B}$ . $c^{HB}$ can be expressed by the following equation:

$$c^{HB}=\frac{c_{o}^{f}c^{H}}{H\left(K+c^{H}\right)}.$$ (38)

The flux of hydrogen ions attached to the solution inside the hydrogel is related to the flux of the hydrogen ions (Chu et al., 1995; Mackie and Meares, 1955)

$${\text{J}}^{HB}=\frac{D^{HB}}{D^{H}}\frac{c^{\mathrm{tot}}}{K+c^{H}}{\text{J}}^{H}$$ (39)

where $D^{HB}$ represents the diffusivity of the hydrogen ions bound to the buffer inside the hydrogel.

By combining Equations 35 and 36 with Equations 37–39, we obtain the following continuity equations for hydrogen ions:

$$\frac{\partial }{\partial t}\left(Hc^{H}+\frac{Hc^{\mathrm{tot}}c^{H}}{K+c^{H}}+\frac{c_{o}^{f}c^{H}}{K+c^{H}}\right)=\nabla \cdot \left({\text{J}}^{H}+\frac{D^{HB}}{D^{H}}\frac{c^{\mathrm{tot}}}{K+c^{H}}{\text{J}}^{H}\right)$$

$$=\nabla \cdot \left(1+\frac{D^{HB}}{D^{H}}\frac{c^{\mathrm{tot}}}{K+c^{H}}\right)\left[\phi^{w}\left(-D^{k}\nabla c^{k}-z^{k}\mu^{k}c^{k}\nabla \psi \right)+c^{k}\nu \right].$$ (40)

The mechanical equations controlling the hydrogel polymer network displacement can be described using the equation of motion in Equation 11, which is reduced to Equation 13 with some simplifications. Considering the swelling process in two dimensions, the components of the stress tensor are.

$$\sigma_{xx}=\frac{E\left(1-\nu \right)}{\left(1+\nu \right)\left(1-2\nu \right)}\frac{\partial u_{x}}{\partial x}+\frac{E\nu }{\left(1+\nu \right)\left(1-2\nu \right)}\frac{\partial u_{y}}{\partial y}-\left(p_{\mathrm{osm}}+p_{\mathrm{elect}}\right)$$ (41)

$$\sigma_{yy}=\frac{E\left(1-\nu \right)}{\left(1+\nu \right)\left(1-2\nu \right)}\frac{\partial u_{y}}{\partial y}+\frac{E\nu }{\left(1+\nu \right)\left(1-2\nu \right)}\frac{\partial u_{x}}{\partial x}-\left(p_{\mathrm{osm}}+p_{\mathrm{elect}}\right)$$ (42)

$$\tau_{xy}=\tau_{yx}=G\left(\frac{\partial u_{x}}{\partial y}+\frac{\partial u_{y}}{\partial x}\right)=\frac{E}{2\left(1+\nu \right)}\left(\frac{\partial u_{x}}{\partial y}+\frac{\partial u_{y}}{\partial x}\right)$$ (43)

where $p_{\mathrm{elect}}$ is the electrostatic stress on the ionic polymer hydrogel. The osmotic pressure, $p_{\mathrm{osm}}$ , is described using Equation 14. The electrostatic stress considered exclusively in this model is given by (Nemat-Nasser and Li, 2000)

$$p_{\mathrm{elect}}=k_{o}\nabla \cdot \left(\varepsilon_{r}\nabla \psi \right)\left[\begin{array}{cc} 1 & 0\\ 0 & 1 \end{array}\right]$$

where $k_{o}$ is a material property that depends on the polymer network geometry and the distribution of fixed charges within the hydrogel.

Bassetti’s model also incorporates the Young’s modulus variation with hydration and an external electric field. The change in the Young’s modulus is given by the following equation (Okay and Durmaz, 2002)

$$E=E_{o}\frac{{\left(H_{o}+1\right)}^{1/3}}{{\left(H+1\right)}^{1/3}}$$

where $E_{o}$ and $H_{o}$ represent the initial Young’s modulus and hydration of the hydrogel, respectively. The relation between the variation of shear modulus ΔG and the Young’s modulus ΔE is (Shiga, 1997)

$$\Delta E=\frac{1}{2\left(1+\nu \right)}\Delta G.$$

**FIGURE 8:**
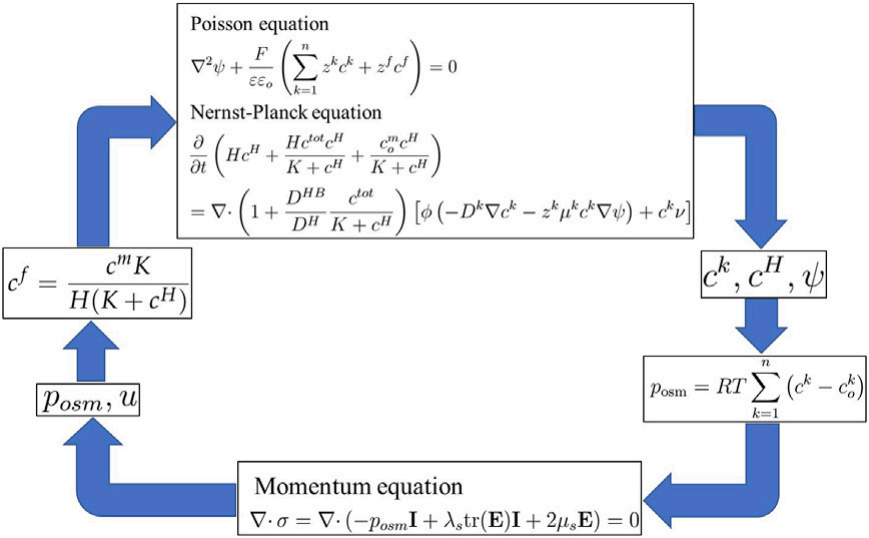
Simulation flow diagram of Bassetti’s transport model.

The following equation provides the change in shear modulus ΔG

$$\Delta G=\frac{9}{4\left(1+H\right)}\varepsilon_{o}\varepsilon_{r}\kappa {\left(\nabla \psi \right)}^{2}$$

where κ is the Clausius-Mossotti function (Jones, 1995), which describes the relative dielectric constant of the polymer network and the solvent. Thus, Equations 1, 40 and 41–43, with the relevant auxiliary equations, constitute Bassetti’s transport model. A complete simulation flow diagram of Bassetti’s transport model (Chatterjee et al., 2003b; a) is shown in Figure 8.

## 6 Porous media theory (PMT)

The PMT (de Boer, 1996) is a macroscopic theory that is an extension of the theory of mixtures (TM) (Mow et al., 1980; Bowen, 1980) using volume fractions (Ehlers, 2009). This theory does not require local porous microstructures or the actual geometric distribution of all the constituents. This theory is a valuable tool for modeling mixtures with immiscible components, indicating that they can be individually identified macroscopically (Acartürk, 2009). Most of the work on stimuli-responsive and, in particular, electroresponsive hydrogels placed in a solution has been performed by Wallmersperger et al. (Attaran, 2017; Sobczyk, 2018).

Continuum-based chemoelectromechanical relations were obtained using Maxwell’s equations, the balance laws of hydrogels, and constitutive relations (Attaran, 2017). The mathematical description includes chemical, electrical, and mechanical field equations combined with the relevant initial, boundary, and jump conditions. This is known as the initial boundary value problem. The complete domain is decomposed into different components, and the domain Λ with boundary ∂Λ is divided into the solution domain S and the hydrogel domain G . The boundary layer inside the solution is ϵ , and ∂ϵ is the reference between the boundary layer and the solution.

### 6.1 Chemical field

The equations for the mobile ions, bound charges, and reference configuration of the chemical field are as follows:

$$\frac{\partial c^{k}}{\partial t}={\left(D_{k}c_{,i}^{k}+z^{k}c^{k}D_{k}\frac{F}{RT}\psi_{,i}\right)}_{,i}\;\text{in}\;\Lambda ;\;\left(k=+,-\right)$$ (44)

$$c_{g}=c_{o}^{g}\left(1-\varepsilon_{ii}\right)\;\text{in}\;G$$ (45)

$${\left(D^{kref}c_{,i}^{kref}\right)}_{,i}=0\;\text{in}\;G\cup \varepsilon$$ (46)

### 6.2 Electric field

The characteristic equations for the mobile ions, bound charges, and reference configuration of the electric field are as follows:

$$\psi_{,ii}=-\frac{F}{\varepsilon_{o}\varepsilon_{r}}\overset{\max}{\underset{k=1}{\sum }}z^{k}c^{k}\;\text{in}\;G$$ (47)

$$\psi_{,ii}=-\frac{F}{\varepsilon_{o}\varepsilon_{r}}\overset{\max}{\underset{k=1}{\sum }}z^{k}c^{k}\;\text{in}\;S$$ (48)

### 6.3 Mechanical field

Finally, the mechanical field equations are given as.

$${\left[C_{\mathrm{ijkl}}\left(\varepsilon_{kl}-g\delta_{kl}RT\underset{\alpha }{\sum }\left(\Delta c^{k}-\Delta c_{o}^{k}\right)\right)\right]}_{,i}=0\;\text{in}\;G$$ (49)

$$\text{where}\varepsilon_{ij}=\frac{1}{2}\left(u_{i,j}+u_{j,i}\right)\;\text{in}\;G$$

$$\Delta c^{k}=c^{k}-c^{kref}\;\text{and}\;\Delta c_{o}^{k}=c_{o}^{k}-c_{o}^{kref}\;\text{in}\;G.$$

Thus, Equations 44–49 collectively constitute the PMT (Attaran et al., 2015; 2018).

## 7 Discussion and comparison

Three different scenarios can be used for the numerical simulation of the chemoelectromechanical equations (Wallmersperger, 2009). The first involves solving each equation separately and then updating the values of the unknowns. This weak-coupling scheme does not converge, and numerous iterations may be required for a single time step. The second scenario involves solving the chemoelectrical fields simultaneously, and then solving the mechanical equation while considering the differential osmotic pressure resulting from the differences in ionic concentrations. It is called sequential, one-way, or semi-coupling, where the mechanical field is usually considered only for the hydrogel domain. In the third case, all three field equations are solved simultaneously; this is referred to as a strong or full coupling scheme. The solution and hydrogel domains were simultaneously modeled chemoelectromechanically; however, the two domains had different material properties.

Doi et al. (1992), and Shiga and Kurauchi (1990), Shiga (1997) were among the pioneers who studied the behavior of electrosensitive hydrogels immersed in solutions under an external electric field. They incorporate Flory and Rehner’s statistical theory (Flory and Rehner, 1943a; b; Flory, 1953) and Donnan’s equilibrium theory (Donnan, 1924). Grimshaw et al. (1990), (1989) provided a macroscopic continuum explanation for the dynamic response of polyelectrolyte hydrogels under an electric field. However, these models are unsuitable for accurately describing the behavior of hydrogels in a solution under an applied electric field. A major contribution to the modeling of electrically stimulated hydrogels depends on the biphasic (Mow et al., 1980), triphasic (Lai et al., 1991), quadphasic (Huyghe and Janssen, 1997), and generalized multiphasic theory (Gu et al., 1998). These theories are relevant, as they were initially proposed to characterize the electrochemical properties of cartilage tissue. Hon et al. (2000), Zhou et al. (2002) used the multiphasic theory to model hydrogels immersed in a solution with an applied electric field. This method has limitations because the computational domain is limited to hydrogel samples.

Subsequently, multiphasic, transport, and porous media theories were reported, which are comprehensive for describing the behavior of electrosensitive hydrogels. All the modeling theories reported in this paper for electrosensitive hydrogels placed in a solution under an external electric field are summarized in Table 1. Initially, these theories were simulated using in-house software, which is not openly available. Subsequently, the simulations were also performed using commercial finite element solvers, i.e., ABAQUS and COMSOL Multiphysics^Ⓡ^. We employed the open-source finite element software, FEniCS (Logg et al., 2012) to solve the Wallmerperger transport model for cartilage tissue engineering (Farooqi et al., 2020). Recently, Mehr and Hatami-Marbini (2022) have used FEniCS to solve a similar chemoelectromechanical model to investigate the electroactive response of scleral tissue (Mehr and Hatami-Marbini, 2022).

**TABLE 1:** Summary of modeling theories for the electrosensitive hydrogels.

Study	Simulated material	Modeling theroy	Coupling scheme	Solution method	Software	Experimental validation	Large deformation	Transient	Geometry	Fixation
Grimshaw et al. (1989), (1990), Grimshaw (1989)	Polymethacrylic acid (PMAA)	Transport	Sequential	Crank-Nicholson	Custom-built	Quantitative	X	✓	1D	Edge
Shiga and Kurauchi (1990), Shiga (1997)	Poly (sodium acrylate) (PAANa)	Transport	NR	NR	Custom-built	Quantitative	X	✓	1D	Edge
Doi et al. (1992)	Acryl acid–acrylamide copolymer	Transport	Sequential	Numerical	Custom-built	Qualitative	X	✓	1D	Edge
Hon et al. (2000)	Chitosan and	Transport	Sequential	Meshless Radial Basis	Custom-built	Quantitative	X	X	1D	Center
Zhou et al. (2002)	poly (ethylene glycol) (PEG)			Function			X	X		
(Li et al., 2004a; c)	Polyelectrolyte hydrogel	Multiphasic Mixture/MECe	Sequential	Meshless Hermite-Cloud	Custom-built	Quantitative	X	X	1D	Center
Chen, 2004; Chen et al. (2005)				Meshless Hermite-Cloud			X	✓	1D	Center
Li et al. (2006)				Meshless Hermite-Cloud			X	✓	1D	Edge
Chen and Ma (2006)				Meshless Finite-Cloud			X	X	2D	Edge
Yuan et al. (2007)				Meshless Hermite-Cloud			X	✓	1D	Center
Li et al., 2007a; Li (2009a)				Meshless Hermite-Cloud			X	✓	1D	Edge
Yuan and Li (2013)				Meshless Hermite-Cloud			X	✓	1D	Center
Wallmersperger (2003)	Polyelectrolyte hydrogel	Wallmersperger’s Transport	Sequential	Finite Element	Custom-built	Qualitative	X	✓	1D	Center
(Wallmersperger et al., 2004a; c)			Sequential		Custom-built		X	X	2D	Center and Edge
Wallmersperger et al. (2004b)			Sequential		Custom-built		X	X	2D	Center
Wallmersperger and Ballhause (2008)			Fully-Coupled		Custom-built		X	✓	1D	Center
Wallmersperger et al. (2008)			Fully-Coupled		Custom-built		X	X	1D	Center
Wallmersperger et al. (2009)			Fully-Coupled		ABAQUS		X	✓	1D	Center
Lam et al. (2006)	Polyelectrolyte hydrogel	Li’s Transport/rMECe	Sequential	Meshless Hermite-Cloud	Custom-built	Quantitative	✓	X	1D	Center
(Li et al., 2007d; c)							✓	X		
Luo et al. (2007a), 2008a							✓	X		
Li et al. (2007b)	Polyelectrolyte hydrogel	Li’s Transport/MECpHe	Sequential	Meshless Hermite-Cloud	Custom-built	Quantitative	✓	X	2D	Center
Luo et al. (2007b), 2008b							✓	X		
Li et al. (2009)							✓	X		
Luo et al. (2009), 2010							✓	X		
Yew et al. (2007)	Polyelectrolyte hydrogel	Li’s Transport/rMECpH-E	Sequential	Meshless Hermite-Cloud	Custom-built	Quantitative	✓	X	1D	Center
Li et al. (2007e)							✓	X		
Ng et al. (2007), 2010							✓	X		
(Chatterjee et al., 2003a; b)	Hydroxyethyl methacrylate (HEMA)	Bassetti’s Transport	Sequential	Meshless Finite-Cloud	Custom-built	Quantitative	X	✓	1D	Center
Bassetti et al. (2005)							X	✓		
(Wallmersperger et al., 2013a; b)	Polyelectrolyte hydrogel	Porous Media Theory	Fully-Coupled	Finite Element	ABAQUS	Qualitative	X	X	1D	Center
Attaran et al. (2015), 2018; Attaran (2017)							X	✓		Edge

Abbreviations: MECe, multi-effect-coupling electric-stimulus model; rMECpH-E, refined multi-effect-coupling pH-electric-stimuli model; rMECe, refined multi-effect-coupling electric-stimulus model; MECpHe, multi-effect-coupling pH-electric-stimuli model; PMT, porous media theory; NR, not recorded; 1D, one-dimension; 2D, two-dimension.

Much work on modeling theories for electrosensitive hydrogels has been conducted by Li Hua’s research group at the National University of Singapore. The group reported a multiphase mixture chemoelectromechanical model called the MECe model and three variants of the transport model, namely, the rMECe, MECpHe, and rMECpH-E models. All these models were solved using the meshless HermiteCloud method (Li et al., 2003) with custom-built software; however, they did not report the details of this software. The MECe model can consider the time-dependent behavior of hydrogels; however, transport models lack this property. However, the evaluation of time-dependent articular cartilage tissue behavior under various conditions provides important insights (Hosseini et al., 2010; Murakami et al., 2021).

Another major contribution to the modeling of hydrogels under electrical stimulation was made by the Wallmersperger group at the Technical University of Dresden, Germany. Initially, they reported the studies where only the electrochemical effect was studied (Wallmersperger et al., 2001; 2002) using the Poisson–Nernst–Planck equations. Subsequently, they coupled it with mechanical field equations to estimate hydrogel deformation (Wallmersperger et al., 2009). This group conducted numerous studies using the transport model and PMT (Wallmersperger et al., 2013a), which were solved using the finite element method. Finite element simulations were performed using custom-built programs and the commercial software ABAQUS. The nonlinear equations of these models were solved numerically using a sequential scheme as well as a fully coupled approach, where all the unknowns were evaluated simultaneously. However, these models cannot simulate large hydrogel deformations at high applied voltages. This is because the linear elastic theory has been used for mechanical deformation. However, the analysis of the large deformation behavior of articular cartilage arising from its unique composition, structure, and nonlinear characteristics is preferred (Woo et al., 1979; Zhang et al., 2015).

Another contribution to the modeling theories of electrosensitive hydrogels is the transport model proposed by Bassetti et al. (2005). This model is based on the studies by De et al. (2002), De and Aluru (2004) initially proposed for modeling pH-sensitive hydrogels. This model incorporates the electrostatic stress of the hydrogel. Unlike transport models, the PMT considers each constituent separately, following the principles of continuum mechanics (Wallmersperger and Leichsenring, 2016). Implementing the PMT can be computationally expensive owing to the number of constituents, but it can simulate transient behavior.

The presented theories were evaluated to determine whether they could simulate large deformations of hydrogels or if they were valid only for small deformations. The possibility of steady-state or transient simulation was also investigated. We also evaluated whether one- or two-dimensional geometries were considered in the simulation domain. It is evident from Table 1 that no theory has been used to simulate three-dimensional geometries. Finally, the hydrogel fixation was evaluated to determine whether the fixation was center- or edge-fixed to avoid rigid-body motion in the mechanical simulation.

A limitation of Wallmersperger’s transport model is that a small deformation is assumed, even at high applied potentials (Luo, 2008), as is evident from the data in Table 1. However, it has been experimentally confirmed that hydrogels undergo large deformations when a high electric potential is applied (Zhou et al., 2002). In addition, the effect of fixed charges was ignored (Li, 2009b). The osmotic pressure calculation in this model depends only on the ionic concentrations. However, electric potential should also be considered when calculating the osmotic pressure with an applied electric field.

In transport models, hydrogel swelling and deformation depend on ionic diffusion, whereas fluid flow inside the hydrogel is ignored. In addition, osmotic pressure instead of fluid pressure was used to calculate the stress of the hydrogel mixture. This osmotic pressure can be determined from the ionic concentration differences between the solution bath and the interior of the hydrogel. Osmotic and fluid pressures are essential parameters for cartilage characterization (Farooq and Siddique, 2021; Warren and Bajpayee, 2022).

The model proposed by Wallmersperger and Ballhause (2008) for electrosensitive hydrogels is approximate and difficult to extend to two- and three-dimensional geometries. The system, which is composed of a hydrogel scaffold placed in a solution with an applied electric field, cannot attain thermodynamic equilibrium even in a steady state. Moreover, the chemical potential of the water in the system was nonuniform. Thus, the osmotic pressure considered in the model proposed by Wallmersperger and Ballhause (2008) is not the same as the fluid pressure. Therefore, it cannot be used as a mechanical parameter for thermodynamic equilibrium (Feng et al., 2011).

An experimental verification of these theories is also provided. While most theories have been compared quantitatively, others have been compared qualitatively. The theoretical transport model proposed by Grimshaw et al. (1990) was experimentally verified by comparing it with the measurements of the chemically and electrically induced swelling and shrinking of crosslinked polymethacrylic acid (PMAA) membranes. Shiga and Kurauchi (1990) qualitatively compared the theoretical deformation behavior under an electric field with the experimental findings of a poly (sodium acrylate) (PAANa) hydrogel placed in a NaOH solution between two electrodes (without touching the electrodes). The numerical results of the transport model by Doi et al. (1992) were qualitatively compared with experimental results focusing on acryl acid–acrylamide copolymer hydrogels (Shiga and Kurauchi, 1990). Zhou et al. (2002) theoretically and experimentally investigated the deformation response of chitosan and poly (ethylene glycol) (PEG) hydrogel strips immersed in an acidic solution under an external electric field. The numerical simulation results of the multiphasic mixture/MECe theory (Li et al., 2004c; a; Chen et al., 2005) were quantitatively compared with the experimental results of the variation of the average PEG hydrogel curvature *versus* the applied voltage (Zhou et al., 2002) and experimentally measured endpoint displacement of the PAANa hydrogel strip at different time steps (Shiga and Kurauchi, 1990). Wallmersperger et al. (2004a), (2004b) qualitatively compared the transport theory results with the experimental results on poly (acrylamide/acrylic acid) (PAAm/PAA) hydrogel under an electric field (Gülch et al., 2000). The numerical results of the rMECe theory involving polyelectrolyte hydrogels (Lam et al., 2006; Luo et al., 2007a; Li et al., 2007d) were quantitatively validated using the experimental results of the PEG hydrogel strip (Zhou et al., 2002). Similarly, the numerical results of the MECpHe theory (Li et al., 2007b; Luo et al., 2007b) were compared with the bending behavior of an interpenetrating polymer network composed of PMAA and a poly (vinyl alcohol) (PVA) hydrogel upon application of an electric field, as reported by Kim et al. (2004) and were observed to be consistent with each other. Li’s transport/rMECpH-E theory (Yew et al., 2007; Li et al., 2007e; Ng et al., 2007) was experimentally verified by comparing it with the results reported in the literature (Zhou et al., 2002; De et al., 2002; Kim et al., 2003; 2004). Bassetti’s transport theory (Bassetti et al., 2005) was quantitatively benchmarked against the experimental results. There is a significant increase in the swelling of electroresponsive hydroxyethyl methacrylate (HEMA) gels with increasing applied voltages across the electrodes (Chatterjee et al., 2003b; Chatterjee, 2003). Finally, the PMT for polyelectrolyte hydrogels (Wallmersperger et al., 2013a) was qualitatively compared with the experimental results reported previously for PAAm/PAA (Gülch et al., 2000) and PAANa (Shiga and Kurauchi, 1990) hydrogels. The nature of the experimental validation of these theories is summarized in Table 1.

## 8 Conclusions and outlook

This review presents complete mathematical formulations of various theories available in the literature to describe the mechanism of electrical stimulation of polyelectrolyte hydrogels under an electric field. The application of these theories as models for cartilage tissue engineering using electrical stimulation has been explained in detail. First, the kinematics, balance laws of continuum chemoelectromechanics, and constitutive equations of the fields involved in each theory are presented. Several other important features, such as the modeling approach, coupling scheme, solution method, and simulation software, are outlined. Moreover, the large or small deformation capability, transient or stationary behavior, geometric dimensions, and fixation type are summarized. All these theories are then compared, and their essential features are described.

The coupled multifield models provide an excellent description of the electrical stimulation of polyelectrolyte gels and can effectively be used for the numerical simulation of cartilage repair implants under electrical stimulation. Wallmersperger’s transport model (Wallmersperger, 2003) is the simplest of all the chemoelectromechanical models because it has the least number of unknowns. However, it has certain disadvantages that limit its use in accurately simulating electrosensitive hydrogels. The multiphasic theory/MECe model (Li et al., 2004c) is the most comprehensive theory that can be used for simulation; however, the number of unknowns involved is the highest compared with all others. It also has the ability to simulate time-dependent behaviors. However, it has the disadvantage of not being applicable to large deformations of hydrogels with high input voltages. The transport models proposed by Lam et al. (2006), Li et al. (2007b), Yew et al. (2007) are the most appropriate choices for implementation. However, the disadvantage of these models is that they cannot simulate time-dependent behavior.

Generally, multidimensional simulations are essential for a comprehensive understanding of electrosensitive hydrogels under electrical stimulation. A significant contribution was made regarding the modeling theories of electrosensitive hydrogels to replicate the experimental procedures. However, the available modeling theories mainly consider hydrogels as general polyelectrolyte materials. Further work is necessary so that a modeling approach can be selected depending on the characteristics of the electrosensitive hydrogels, for example, natural *versus* synthetic, macromolecular *versus* supramolecular, conjugated polymers *versus* hydrogels with nanofillers, or the choice of models based on the physical or chemical crosslinking of the hydrogel. To achieve this, modeling studies on the microscale characterization of electrosensitive hydrogels are necessary (Bassetti et al., 2005).

## Footnotes

1 The readers not familiar with basic tensor theory can have a look at some relevant book, e.g., (Zohdi, 2017).

## References

[B1] Acartürk A. (2009). Simulation of charged hydrated porous materials. Ph.D. thesis. Stuttgart: University of Stuttgart.

[B2] Ali I. Xudong L. Xiaoqing C. Zhiwei J. Pervaiz M. Weimin Y. (2019). A review of electro-stimulated gels and their applications: present state and future perspectives. Mater. Sci. Eng. C 103, 109852. 10.1016/j.msec.2019.109852 31349434

[B3] Amiryaghoubi N. Fathi M. Barar J. Noroozi-Pesyan N. Omidian H. Omidi Y. (2023). Application of graphene in articular cartilage tissue engineering and chondrogenic differentiation. J. Drug Deliv. Sci. Technol. 83, 104437. 10.1016/j.jddst.2023.104437

[B4] Attaran A. (2017). Modeling and numerical simulation of electroactive and magnetoactive polymer gels. Ph.D. thesis. Dresden: Technical University Dresden.

[B5] Attaran A. Brummund J. Wallmersperger T. (2015). Modeling and simulation of the bending behavior of electrically-stimulated cantilevered hydrogels. Smart Mater. Struct. 24, 035021. 10.1088/0964-1726/24/3/035021

[B6] Attaran A. Keller K. Wallmersperger T. (2018). Modeling and simulation of hydrogels for the application as finger grippers. J. Intelligent Material Syst. Struct. 29, 371–387. 10.1177/1045389X17708040

[B7] Bai X. Sun H. Jia L. Xu J. Zhang P. Zhang D. (2023). Chondrocyte targeting gold nanoparticles protect growth plate against inflammatory damage by maintaining cartilage balance. Mater. Today Bio 23, 100795. 10.1016/j.mtbio.2023.100795 PMC10519832 37766899

[B8] Bassetti M. J. Chatterjee A. N. Aluru N. R. Beebe D. J. (2005). Development and modeling of electrically triggered hydrogels for microfluidic applications. J. Microelectromechanical Syst. 14, 1198–1207. 10.1109/jmems.2005.845407

[B9] Bhosale A. M. Richardson J. B. (2008). Articular cartilage: structure, injuries and review of management. Br. Med. Bull. 87, 77–95. 10.1093/bmb/ldn025 18676397

[B10] Biot M. A. (1956). Theory of deformation of a porous viscoelastic anisotropic solid. J. Appl. Phys. 27, 459–467. 10.1063/1.1722402

[B11] Bowen R. M. (1980). Incompressible porous media models by use of the theory of mixtures. Int. J. Eng. Sci. 18, 1129–1148. 10.1016/0020-7225(80)90114-7

[B12] Brighton C. T. Wang W. Clark C. C. (2006). Up-regulation of matrix in bovine articular cartilage explants by electric fields. Biochem. Biophys. Res. Commun. 342, 556–561. 10.1016/j.bbrc.2006.01.171 16487926

[B13] Brighton T. Wang W. Clark C. C. (2008). The effect of electrical fields on gene and protein expression in human osteoarthritic cartilage explants. J. Bone Jt. Surg. 90, 833–848. 10.2106/jbjs.f.01437 18381322

[B14] Buschmann M. D. Grodzinsky A. J. (1995). A molecular model of proteoglycan-associated electrostatic forces in cartilage mechanics. J. Biomechanical Eng. 117, 179–192. 10.1115/1.2796000 7666655

[B15] Carayon I. Gaubert A. Mousli Y. Philippe B. (2020). Electro-responsive hydrogels: macromolecular and supramolecular approaches in the biomedical field. Biomaterials Sci. 8, 5589–5600. 10.1039/d0bm01268h 32996479

[B16] Chandrasekharaiah D. S. Debnath L. (1994). Continuum mechanics. San Diego: Academic Press. 10.1016/C2009-0-21209-8

[B17] Chao P.-H. G. Roy R. Mauck R. L. Liu W. Valhmu W. B. Hung C. T. (2000). Chondrocyte translocation response to direct current electric fields. J. Biomechanical Eng. 122, 261–267. 10.1115/1.429661 10923294

[B18] Chatterjee A. N. (2003). Modeling and simulation of polymeric hydrogels and hydrogel based devices for MEMS/Bio-MEMS applications. Ph.D. thesis. Urbana, Illinois: University of Illinois at Urbana-Champaign.

[B19] Chatterjee A. N. De S. K. Aluru N. (2003a). “Electrically triggered hydrogels: mathematical models and simulations,” in 2003 nanotechnology conference and trade show-nanotech 2003, Cambridge, Massachusetts: TechConnect. 130–133.

[B20] Chatterjee A. N. Yu Q. Moore J. S. Aluru N. R. (2003b). Mathematical modeling and simulation of dissolvable hydrogels. J. Aerosp. Eng. 16, 55–64. 10.1061/(ASCE)0893-1321(2003)16:2(55)

[B21] Chen J. (2004). Multiphasic model development and meshless simulations of electric-sensitive hydrogels. Ph.D. thesis. Singapore: National University of Singapore.

[B22] Chen J. Ma G. (2006). Modelling deformation behaviour of polyelectrolyte gels under chemo-electro-mechanical coupling effects. Int. J. Numer. Methods Eng. 68, 1052–1071. 10.1002/nme.1752

[B23] Chen J. Li H. Lam K. Y. (2005). Transient simulation for kinetic responsive behaviors of electric-sensitive hydrogels subject to applied electric field. Mater. Sci. Eng. C 25, 710–712. 10.1016/j.msec.2005.06.020

[B24] Cheng S. Yang J. Song J. Cao X. Zhou B. Yang L. (2025). A motion-responsive injectable lubricative hydrogel for efficient achilles tendon adhesion prevention. Mater. Today Bio 30, 101458. 10.1016/j.mtbio.2025.101458 PMC11762619 39866793

[B25] Chu Y. Varanasi P. P. McGlade M. J. Varanasi S. (1995). pH-Induced swelling kinetics of polyelectrolyte hydrogels. J. Appl. Polym. Sci. 58, 2161–2176. 10.1002/app.1995.070581203

[B26] Culma J. J. S. Morales J. M. G. Uribe Y. A. H. Garzón-Alvarado D. A. Leal-Marin S. Glasmacher B. (2025). Effects of electric fields on the modulation of chondrocytes dynamics in gelatin scaffolds: a novel approach to optimize cartilage tissue engineering. J. Biomaterials Sci. Polym. Ed., 1–20. 10.1080/09205063.2025.2466971 39998819

[B27] De S. K. Aluru N. R. (2004). A chemo-electro-mechanical mathematical model for simulation of pH sensitive hydrogels. Mech. Mater. 36, 395–410. 10.1016/S0167-6636(03)00067-X

[B28] de Boer R. (1996). Highlights in the historical development of the porous media theory: toward a consistent macroscopic theory. Appl. Mech. Rev. 49, 201–262. 10.1115/1.3101926

[B29] De S. K. Aluru N. R. Johnson B. Crone W. C. Beebe D. J. Moore J. S. (2002). Equilibrium swelling and kinetics of pH-responsive hydrogels: models, experiments, and simulations. J. Microelectromechanical Syst. 11, 544–555. 10.1109/JMEMS.2002.803281

[B30] Doi M. Matsumoto M. Hirose Y. (1992). Deformation of ionic polymer gels by electric fields. Macromolecules 25, 5504–5511. 10.1021/ma00046a058

[B31] Donnan F. G. (1924). The theory of membrane equilibria. Chem. Rev. 1, 73–90. 10.1021/cr60001a003

[B32] Ehlers W. (2002). “Foundations of multiphasic and porous materials,” in Porous media: theory, experiments and numerical applications (Berlin, Heidelberg: Springer), 3–86.

[B33] Ehlers W. (2009). Challenges of porous media models in geo- and biomechanical engineering including electro-chemically active polymers and gels. Int. J. Adv. Eng. Sci. Appl. Math. 1, 1–24. 10.1007/s12572-009-0001-z

[B34] El-Husseiny H. M. Mady E. A. Hamabe L. Abugomaa A. Shimada K. Yoshida T. (2022). Smart/stimuli-responsive hydrogels: cutting-edge platforms for tissue engineering and other biomedical applications. Mater. Today Bio 13, 100186. 10.1016/j.mtbio.2021.100186 PMC8669385 34917924

[B35] Erol O. Pantula A. Liu W. Gracias D. H. (2019). Transformer hydrogels: a review. Adv. Mater. Technol. 4, 1900043–27. 10.1002/admt.201900043

[B36] Esfandiari E. Roshankhah S. Mardani M. Hashemibeni B. Naghsh E. Kazemi M. (2014). The effect of high frequency electric field on enhancement of chondrogenesis in human adipose-derived stem cells. Iran. J. Basic Med. Sci. 17, 571–576. 10.22038/ijbms.2014.3188 25422749 PMC4240790

[B37] Farooq U. Siddique J. (2021). Compressive stress relaxation behavior of articular cartilage and its effects on fluid pressure and solid displacement due to non-newtonian flow. Comput. Methods Biomechanics Biomed. Eng. 24, 161–172. 10.1080/10255842.2020.1817408 33017177

[B38] Farooqi A. R. (2020). Computational modeling of electroactive hydrogels for cartilage – tissue repair using electrical stimulation. Ph.D. thesis. Rostock: University of Rostock.

[B39] Farooqi A. R. Bader R. van Rienen U. (2019a). Numerical study on electromechanics in cartilage tissue with respect to its electrical properties. Tissue Eng. Part B Rev. 25, 152–166. 10.1089/ten.teb.2018.0214 30351244 PMC6486674

[B40] Farooqi A. R. Zimmermann J. Bader R. van Rienen U. (2019b). Numerical simulation of electroactive hydrogels for cartilage – tissue engineering. Materials 12, 2913. 10.3390/ma12182913 31505797 PMC6774344

[B41] Farooqi A. R. Zimmermann J. Bader R. van Rienen U. (2020). Computational study on electromechanics of electroactive hydrogels for cartilage-tissue repair. Comput. Methods Programs Biomed. 197, 105739. 10.1016/j.cmpb.2020.105739 32950923

[B42] Feng L. Jia Y. Li X. An L. (2011). Comparison of the multiphasic model and the transport model for the swelling and deformation of polyelectrolyte hydrogels. J. Mech. Behav. Biomed. Mater. 4, 1328–1335. 10.1016/j.jmbbm.2011.05.001 21783142

[B43] Flory P. J. (1953). Principles of polymer chemistry. Ithaca, New York: Cornell University Press.

[B44] Flory P. J. Rehner J. (1943a). Statistical mechanics of cross-linked polymer networks i. rubberlike elasticity. J. Chem. Phys. 11, 512–520. 10.1063/1.1723791

[B45] Flory P. J. Rehner J. (1943b). Statistical mechanics of cross-linked polymer networks ii. swelling. J. Chem. Phys. 11, 521–526. 10.1063/1.1723792

[B46] Frank E. H. Grodzinsky A. J. (1987a). Cartilage electromechanics-i. electrokinetic transduction and the effects of electrolyte ph and ionic strength. J. Biomechanics 20, 615–627. 10.1016/0021-9290(87)90282-X 3611137

[B47] Frank E. H. Grodzinsky A. J. (1987b). Cartilage electromechanics-ii. a continuum model of cartilage electrokinetics and correlation with experiments. J. Biomechanics 20, 629–639. 10.1016/0021-9290(87)90283-1 3611138

[B48] Gaspar V. M. Lavrador P. Borges J. Oliveira M. B. Mano J. F. (2020). Advanced bottom-up engineering of living architectures. Adv. Mater. 32, 1903975. 10.1002/adma.201903975 31823448

[B49] Grimshaw P. E. (1989). Electrical control of solute transport across polyelectrolyte membranes. Ph.D. thesis. Cambridge, MA: Massachusetts Institute of Technology.

[B50] Grimshaw P. E. Grodzinsky A. J. Yarmush M. L. Yarmush D. M. (1989). Dynamic membranes for protein transport: chemical and electrical control. Chem. Eng. Sci. 44, 827–840. 10.1016/0009-2509(89)85256-X

[B51] Grimshaw P. E. Nussbaum J. H. Grodzinsky A. J. Yarmush M. L. (1990). Kinetics of electrically and chemically induced swelling in polyelectrolyte gels. J. Chem. Phys. 93, 4462–4472. 10.1063/1.458729

[B52] Gu W. Y. Lai W. M. Mow V. C. (1998). A mixture theory for charged-hydrated soft tissues containing multi-electrolytes: passive transport and swelling behaviors. J. Biomechanical Eng. 120, 169–180. 10.1115/1.2798299 10412377

[B53] Gülch R. W. Holdenried J. Weible A. Wallmersperger T. Kröplin B. (2000). “Polyelectrolyte gels in electric fields: a theoretical and experimental approach,” Proceedings of SPIE, Bellingham, Washington, March 6-9 2000. 3987, 193–202. 10.1117/12.387778

[B54] Hashemi-Afzal F. Fallahi H. Bagheri F. Collins M. N. Eslaminejad M. B. Seitz H. (2025). Advancements in hydrogel design for articular cartilage regeneration: a comprehensive review. Bioact. Mater. 43, 1–31. 10.1016/j.bioactmat.2024.09.005 39318636 PMC11418067

[B55] Hatami-Marbini H. Mehr J. A. (2022). Modeling and experimental investigation of electromechanical properties of scleral tissue; a CEM model using an anisotropic hyperelastic constitutive relation. Biomechanics Model. Mechanobiol. 21, 1325–1337. 10.1007/s10237-022-01590-5 35962249

[B56] He W. Kienzle A. Liu X. Müller W. E. Feng Q. (2015). *In vitro* 30 nm silver nanoparticles promote chondrogenesis of human mesenchymal stem cells. RSC Adv. 5, 49809–49818. 10.1039/c5ra06386h

[B57] He W. Wang Y. Li X. Ji Y. Yuan J. Yang W. (2024). Sealing the pandora’s vase of pancreatic fistula through entrapping the digestive enzymes within a dextrorotary (d)-peptide hydrogel. Nat. Commun. 15, 7235. 10.1038/s41467-024-51734-7 39174548 PMC11341566

[B58] Helfferich F. (1962). Ion exchange. New York, NY: McGraw-Hill Book Company, Inc.

[B59] Hiemer B. Krogull M. Bender T. Ziebart J. Krueger S. Bader R. (2018). Effect of electric stimulation on human chondrocytes and mesenchymal stem cells under normoxia and hypoxia. Mol. Med. Rep. 18, 2133–2141. 10.3892/mmr.2018.9174 29916541 PMC6072227

[B60] Hon Y. C. Lu M. W. Xue W. M. Zhou X. (1999). A new formulation and computation of the triphasic model for mechano-electrochemical mixtures. Comput. Mech. 24, 155–165. 10.1007/s004660050448

[B61] Hon Y. C. Lu M. W. Mak A. F. T. Zhou X. (2000). “Mechano-electrochemical response analysis of a hydrogel strip under electric field,” in Advances in computational engineering and sciences (Palmdale, California: Tch Science Press), 1681–1686.

[B62] Horkay F. Tasaki I. Basser P. J. (2000). Osmotic swelling of polyacrylate hydrogels in physiological salt solutions. Biomacromolecules 1, 84–90. 10.1021/bm9905031 11709847

[B63] Hosseini A. Van de Velde S. K. Kozanek M. Gill T. J. Grodzinsky A. J. Rubash H. E. (2010). *In-vivo* time-dependent articular cartilage contact behavior of the tibiofemoral joint. Osteoarthr. Cartil. 18, 909–916. 10.1016/j.joca.2010.04.011 PMC2900485 20434573

[B64] Hosseini F. S. Saburi E. Enderami S. E. Ardeshirylajimi A. Bagherabad M. B. Marzouni H. Z. (2019). Improved chondrogenic response of mesenchymal stem cells to a polyethersulfone/polyaniline blended nanofibrous scaffold. J. Cell. Biochem. 120, 11358–11365. 10.1002/jcb.28412 30746743

[B65] Hu L. Zhang Q. Li X. Serpe M. J. (2019). Stimuli-responsive polymers for sensing and actuation. Mater. Horizons 6, 1774–1793. 10.1039/c9mh00490d

[B66] Huyghe J. M. Janssen J. D. (1997). Quadriphasic mechanics of swelling incompressible porous media. Int. J. Eng. Sci. 35, 793–802. 10.1016/S0020-7225(96)00119-X

[B67] Jackson A. R. Gu W. Y. (2009). Transport properties of cartilaginous tissues. Curr. Rheumatol. Rep. 5, 40–50. 10.2174/157339709787315320 PMC2748424 20126303

[B68] Jones T. B. (1995). Electromechanics of particles. New York: Cambridge University Press.

[B69] Kaith B. S. Singh A. Sharma A. K. Sud D. (2021). Hydrogels: synthesis, classification, properties and potential applications—A brief review. J. Polym. Environ. 29, 3827–3841. 10.1007/s10924-021-02184-5

[B70] Kanaan A. F. Piedade A. P. (2022). Electro-responsive polymer-based platforms for electrostimulation of cells. Mater. Adv. 3, 2337–2353. 10.1039/d1ma01012c

[B71] Kim S. J. Lee K. J. Kim S. I. Lee Y. M. Chung T. D. Lee S. H. (2003). Electrochemical behavior of an interpenetrating polymer network hydrogel composed of poly(propylene glycol) and poly(acrylic acid). J. Appl. Polym. Sci. 89, 2301–2305. 10.1002/app.12327

[B72] Kim S. J. Yoon S. G. Lee S. M. Lee S. H. Kim S. I. (2004). Electrical sensitivity behavior of a hydrogel composed of polymethacrylic acid/poly(vinyl alcohol). J. Appl. Polym. Sci. 91, 3613–3617. 10.1002/app.13597

[B73] Krueger S. Riess A. Jonitz-Heincke A. Weizel A. Seyfarth A. Seitz H. (2021). Establishment of a new device for electrical stimulation of non-degenerative cartilage cells *in vitro* . Int. J. Mol. Sci. 22, 394–397. 10.3390/ijms22010394 33401406 PMC7794805

[B74] Lai W. M. Hou J. S. Mow V. C. (1991). A triphasic theory for the swelling and deformation behaviors of articular cartilage. J. Biomechanical Eng. 113, 245–258. 10.1115/1.2894880 1921350

[B75] Lai W. M. Mow V. C. Sun D. D. Ateshian G. A. (2000). On the electric potentials inside a charged soft hydrated biological tissue: streaming potential *versus* diffusion potential. J. Biomechanical Eng. 122, 336–346. 10.1115/1.1286316 11036556

[B76] Lam K. Y. Li H. Ng T. Y. Luo R. (2006). Modeling and simulation of the deformation of multi-state hydrogels subjected to electrical stimuli. Eng. Analysis Bound. Elem. 30, 1011–1017. 10.1016/j.enganabound.2006.03.011

[B77] Lavrador P. Esteves M. R. Gaspar V. M. Mano J. F. (2021). Stimuli-responsive nanocomposite hydrogels for biomedical applications. Adv. Funct. Mater. 31, 2005941. 10.1002/adfm.202005941

[B78] Lei Y. Zhang Q. Kuang G. Wang X. Fan Q. Ye F. (2022). Functional biomaterials for osteoarthritis treatment: from research to application. Smart Med. 1, e20220014–e20220014. 10.1002/smmd.20220014 39188730 PMC11235767

[B79] Li H. (2009a). Kinetics of smart hydrogels responding to electric field: a transient deformation analysis. Int. J. Solids Struct. 46, 1326–1333. 10.1016/j.ijsolstr.2008.11.001

[B80] Li H. (2009b). Smart hydrogel modelling. 1 edn. Singapore Berlin, Heidelberg: Springer.

[B81] Li H. Ng T. Y. Cheng J. Q. Lam K. Y. (2003). Hermite-Cloud: a novel true meshless method. Comput. Mech. 33, 30–41. 10.1007/s00466-003-0497-1

[B82] Li H. Chen J. Lam K. Y. (2004a). Multiphysical modeling and meshless simulation of electric-sensitive hydrogels. J. Polym. Sci. Part B Polym. Phys. 42, 1514–1531. 10.1002/polb.20025

[B83] Li H. Yew Y. K. Lam K. Y. Ng T. Y. (2004b). Numerical simulation of pH-stimuli responsive hydrogel in buffer solutions. Colloids Surfaces A Physicochem. Eng. Aspects 249, 149–154. 10.1016/j.colsurfa.2004.08.068

[B84] Li H. Yuan Z. Lam K. Y. Lee H. P. Chen J. Hanes J. (2004c). Model development and numerical simulation of electric-stimulus-responsive hydrogels subject to an externally applied electric field. Biosens. Bioelectron. 19, 1097–1107. 10.1016/j.bios.2003.10.004 15018965

[B85] Li H. Ng T. Y. Yew Y. K. Lam K. Y. (2005). Modeling and simulation of the swelling behavior of ph-stimulus-responsive hydrogels. Biomacromolecules 6, 109–120. 10.1021/bm0496458 15638511

[B86] Li H. Chen J. Lam K. Y. (2006). A transient simulation to predict the kinetic behavior of hydrogels responsive to electric stimulus. Biomacromolecules 7, 1951–1959. 10.1021/bm060064n 16768419

[B87] Li H. Chen J. Lam K. Y. (2007a). Transient simulation of kinetics of electric-sensitive hydrogels. Biosens. Bioelectron. 22, 1633–1641. 10.1016/j.bios.2006.07.016 16930980

[B88] Li H. Luo R. Birgersson E. Lam K. Y. (2007b). Modeling of multiphase smart hydrogels responding to pH and electric voltage coupled stimuli. J. Appl. Phys. 101, 114905. 10.1063/1.2736862

[B89] Li H. Luo R. Lam K. Y. (2007c). Modeling and simulation of deformation of hydrogels responding to electric stimulus. J. Biomechanics 40, 1091–1098. 10.1016/j.jbiomech.2006.04.012 16780849

[B90] Li H. Luo R. Lam K. Y. (2007d). Modeling of ionic transport in electric-stimulus-responsive hydrogels. J. Membr. Sci. 289, 284–296. 10.1016/j.memsci.2006.12.011

[B91] Li H. Ng T. Y. Yew Y. K. Lam K. Y. (2007e). Meshless modeling of pH-sensitive hydrogels subjected to coupled pH and electric field stimuli: young modulus effects and case studies. Macromol. Chem. Phys. 208, 1137–1146. 10.1002/macp.200600620

[B92] Li H. Luo R. Lam K. Y. (2009). Multiphysics modeling of electrochemomechanically smart microgels responsive to coupled pH/electric stimuli. Macromol. Biosci. 9, 287–297. 10.1002/mabi.200800139 19009512

[B93] Liu H. Chen J. Qiao S. Zhang W. (2021). Carbon-based nanomaterials for bone and cartilage regeneration: a review. ACS Biomaterials Sci. Eng. 7, 4718–4735. 10.1021/acsbiomaterials.1c00759 34586781

[B94] Liu C. T. Yu J. Lin M. H. Chang K. H. Lin C. Y. Cheng N. C. (2023). Biophysical electrical and mechanical stimulations for promoting chondrogenesis of stem cells on pedot:pss conductive polymer scaffolds. Biomacromolecules 24, 3858–3871. 10.1021/acs.biomac.3c00506 37523499

[B95] Liu C. Zhao L. Dong H. Hua Z. Wang Y. Wang Y. (2025). Experimental investigation on the reverse mechano-electrical effect of porcine articular cartilage. Front. Bioeng. Biotechnol. 13, 1485593. 10.3389/fbioe.2025.1485593 39963171 PMC11830689

[B96] Logg A. Mardal K.-A. Wells G. (2012). Automated solution of differential equations by the finite element method: the FEniCS book, 84. Berlin, Heidelberg: Springer Science and Business Media.

[B97] Luo R. (2008). Modeling and simulations of equilibrium of environmentally sensitive hydrogels. Ph.D. thesis. Palmdale, California: National University of Singapore.

[B98] Luo R. Li H. Lam K. Y. (2007a). Coupled chemo-electro-mechanical simulation for smart hydrogels that are responsive to an external electric field. Smart Mater. Struct. 16, 1185–1191. 10.1088/0964-1726/16/4/029

[B99] Luo R. Li H. Lam K. Y. (2007b). Modeling and simulation of chemo-electro-mechanical behavior of ph-electric-sensitive hydrogel. Anal. Bioanal. Chem. 389, 863–873. 10.1007/s00216-007-1483-9 17643229

[B100] Luo R. Li H. Birgersson E. Khin Y. L. (2008a). Modeling of electric-stimulus-responsive hydrogels immersed in different bathing solutions. J. Biomed. Mater. Res. - Part A 85, 248–257. 10.1002/jbm.a.31586 17688273

[B101] Luo R. Li H. Lam K. Y. (2008b). Modeling and analysis of ph-electric-stimuli-responsive hydrogels. J. Biomaterials Sci. Polym. Ed. 19, 1597–1610. 10.1163/156856208786440532 19017473

[B102] Luo R. Li H. Ng T. Y. (2009). Analysis of ionic transport interaction between soft smart hydrogel and solution in BioMEMS channel. Adv. Mater. Res. 74, 125–128. 10.4028/www.scientific.net/AMR.74.125

[B103] Luo R. Li H. Ng T. Y. Lam K. Y. (2010). Computational analysis of influence of ionic strength on smart hydrogel subject to coupled pH-electric environmental stimuli. Mech. Adv. Mater. Struct. 17, 573–583. 10.1080/15376490903556568

[B104] MacGinitie L. A. Gluzband Y. A. Grodzinsky A. J. (1994). Electric field stimulation can increase protein synthesis in articular cartilage explants. J. Orthop. Res. 12, 151–160. 10.1002/jor.1100120202 8164086

[B105] Mackie J. S. Meares P. (1955). The diffusion of electrolytes in a cation-exchange resin membrane. I. Theoretical. Proc. R. Soc. A Math. Phys. Eng. Sci. 232, 498–509. 10.1098/rspa.1955.0234

[B106] Mehr J. A. Hatami-Marbini H. (2022). Experimental and numerical analysis of electroactive characteristics of scleral tissue. Acta Biomater. 143, 127–137. 10.1016/j.actbio.2022.01.017 35038585

[B107] Mehr J. A. Hatami-Marbini H. (2023). Finite deformation of scleral tissue under electrical stimulation: an arbitrary lagrangian-eulerian finite element method. Bioengineering 10, 920. 10.3390/bioengineering10080920 37627805 PMC10451613

[B108] Miguel F. Barbosa F. Ferreira F. C. Silva J. C. (2022). Electrically conductive hydrogels for articular cartilage tissue engineering. Gels 8, 710. 10.3390/gels8110710 36354618 PMC9689960

[B109] Mohamed M. A. Fallahi A. El-Sokkary A. M. A. Salehi S. Akl M. A. Jafari A. (2019). Stimuli-responsive hydrogels for manipulation of cell microenvironment: from chemistry to biofabrication technology. Prog. Polym. Sci. 98, 101147. 10.1016/j.progpolymsci.2019.101147 36467305 PMC9718481

[B110] Mow V. C. Kuei S. C. Lai W. M. Armstrong C. G. (1980). Biphasic creep and stress relaxation of articular cartilage in compression: theory and experiments. J. Biomechanical Eng. 102, 73–84. 10.1115/1.3138202 7382457

[B111] Murakami T. Sakai N. Yarimitsu S. Nakashima K. Yamaguchi T. Sawae Y. (2021). Evaluation of influence of changes in permeability with aging on friction and biphasic behaviors of artificial hydrogel cartilage. Biotribology 26, 100178. 10.1016/j.biotri.2021.100178

[B112] Nemat-Nasser S. (2002). Micromechanics of actuation of ionic polymer-metal composites. J. Appl. Phys. 92, 2899–2915. 10.1063/1.1495888

[B113] Nemat-Nasser S. Li J. Y. (2000). Electromechanical response of ionic polymer-metal composites. J. Appl. Phys. 87, 3321–3331. 10.1063/1.372343

[B114] Nernst W. (1888). Zur Kinetik der in Lösung befindlichen Körper. Z. für Phys. Chem. 2U, 613–637. 10.1515/zpch-1888-0274

[B115] Nernst W. (1889). Die elektromotorische Wirksamkeit der Jonen. Z. für Phys. Chem. 4U, 129–181. 10.1515/zpch-1889-0412

[B116] Ng T. Y. Li H. Yew Y. K. Lam K. Y. (2007). Effects of initial-fixed charge density on pH-sensitive hydrogels subjected to coupled pH and electric field stimuli: a meshless analysis. J. Biomechanical Eng. 129, 148–155. 10.1115/1.2472370 17408319

[B117] Ng T. Y. Li H. Yew Y. K. (2010). Computational analysis of smart soft hydrogels subjected to pH-electrical coupled stimuli: effects of initial geometry. Int. J. Solids Struct. 47, 614–623. 10.1016/j.ijsolstr.2009.10.024

[B118] Ngo L. Knothe Tate M. L. (2020). Osteoarthritis: new strategies for transport and drug delivery across length scales. ACS Biomaterials Sci. Eng. 6, 6009–6020. 10.1021/acsbiomaterials.0c01081 33449636

[B119] Ni X. Xing X. Deng Y. Li Z. (2023). Applications of stimuli-responsive hydrogels in bone and cartilage regeneration. Pharmaceutics 15, 982. 10.3390/pharmaceutics15030982 36986842 PMC10056098

[B120] Nussbaum J. H. (1986). Electric field control of mechanical and electrochemical properties of polyelectrolyte gel membranes. Ph.D. thesis. Cambridge, MA: Massachusetts Institute of Technology.

[B121] Okay O. Durmaz S. (2002). Charge density dependence of elastic modulus of strong polyelectrolyte hydrogels. Polymer 43, 1215–1221. 10.1016/S0032-3861(01)00723-6

[B122] Planck M. (1890). Ueber die Erregung von Electricität und Wärme in Electrolyten. Ann. Phys. 275, 161–186. 10.1002/andp.18902750202

[B123] Qureshi D. Nayak S. K. Maji S. Anis A. Kim D. Pal K. (2019). Environment sensitive hydrogels for drug delivery applications. Eur. Polym. J. 120, 109220. 10.1016/j.eurpolymj.2019.109220

[B124] Rogers Z. J. Zeevi M. P. Koppes R. Bencherif S. A. (2020). Electroconductive hydrogels for tissue engineering: current status and future perspectives. Bioelectricity 2, 279–292. 10.1089/BIOE.2020.0025 34476358 PMC8370338

[B125] Romischke J. Scherkus A. Saemann M. Krueger S. Bader R. Kragl U. (2022). Swelling and mechanical characterization of polyelectrolyte hydrogels as potential synthetic cartilage substitute materials. Gels 8, 296. 10.3390/gels8050296 35621594 PMC9141488

[B126] Roy A. Manna K. Pal S. (2022). Recent advances in various stimuli-responsive hydrogels: from synthetic designs to emerging healthcare applications. Mater. Chem. Front. 6, 2338–2385. 10.1039/d2qm00469k

[B127] Ryan C. N. M. Doulgkeroglou M. N. Zeugolis D. I. (2021). Electric field stimulation for tissue engineering applications. BMC Biomed. Eng. 3, 1–9. 10.1186/s42490-020-00046-0 33397515 PMC7784019

[B128] Saunders J. R. (2013). AC frequency-based cyclical electrical stimulation of hydrogel microactuators. Ph.D. thesis. Edmonton, Alberta: University of Alberta.

[B129] Saunders J. R. Abu-Salih S. Khaleque T. Hanula S. Moussa W. A. (2008). Modeling theories of intelligent hydrogel polymers. J. Comput. Theor. Nanosci. 5, 1942–1960. 10.1166/jctn.2008.1001

[B130] Shi T. Lu H. Zhu J. Zhou X. He C. Li F. (2023). Naturally derived dual dynamic crosslinked multifunctional hydrogel for diabetic wound healing. Compos. Part B Eng. 257, 110687. 10.1016/j.compositesb.2023.110687

[B131] Shiga T. (1997). “Deformation and viscoelastic behavior of polymer gels in electric fields,” in Neutron spin echo spectroscopy viscoelasticity rheology (Berlin, Heidelberg: Springer), 131–163.

[B132] Shiga T. Kurauchi T. (1990). Deformation of polyelectrolyte gels under the influence of electric field. J. Appl. Polym. Sci. 39, 2305–2320. 10.1002/app.1990.070391110

[B133] Sobczyk M. (2018). Modellierung und Simulation von Hydrogelen und hydrogelbasierten Schichtsystemen. Ph.D. thesis. Dresden: Technical University Dresden.

[B134] Suthar K. J. Ghantasala M. K. Mancini D. C. (2007). Simulation of hydrogel micro-actuation. Proc. SPIE 6798, Microelectron. Des. Technol. Packag. III, 67980P–9. 10.1117/12.769925

[B135] Tanaka T. Hocker L. O. Benedek G. B. (1973). Spectrum of light scattered from a viscoelastic gel. J. Chem. Phys. 59, 5151–5159. 10.1063/1.1680734

[B136] Uzieliene I. Popov A. Lisyte V. Kugaudaite G. Bialaglovyte P. Vaiciuleviciute R. (2023). Stimulation of chondrocyte and bone marrow mesenchymal stem cell chondrogenic response by polypyrrole and polypyrrole/gold nanoparticles. Polymers 15, 2571. 10.3390/polym15112571 37299369 PMC10255679

[B137] Vaca-González J. J. Escobar J. F. Guevara J. M. Hata Y. A. Gallego Ferrer G. Garzón-Alvarado D. A. (2019). Capacitively coupled electrical stimulation of rat chondroepiphysis explants: a histomorphometric analysis. Bioelectrochemistry 126, 1–11. 10.1016/j.bioelechem.2018.11.004 30471483

[B138] Vaca-González J. J. Clara-Trujillo S. Guillot-Ferriols M. Ródenas-Rochina J. Sanchis M. J. Ribelles J. L. G. (2020). Effect of electrical stimulation on chondrogenic differentiation of mesenchymal stem cells cultured in hyaluronic acid – Gelatin injectable hydrogels. Bioelectrochemistry 134, 107536. 10.1016/j.bioelechem.2020.107536 32335352

[B139] Van Gelder P. Audenaert E. Calders P. Leybaert L. (2023). A new look at osteoarthritis: threshold potentials and an analogy to hypocalcemia. Front. Aging 4, 977426–6. 10.3389/fragi.2023.977426 36970729 PMC10031104

[B140] Walker B. W. Lara R. P. Mogadam E. Yu C. H. Kimball W. Annabi N. (2019). Rational design of microfabricated electroconductive hydrogels for biomedical applications. Prog. Polym. Sci. 92, 135–157. 10.1016/j.progpolymsci.2019.02.007 32831422 PMC7441850

[B141] Wallmersperger T. (2003). Modellierung und Simulation stimulierbarer polyelektrolytischer Gele. Ph.D. thesis. Düsseldorf: VDI Verlag.

[B142] Wallmersperger T. (2009). “Modelling and simulation of the chemo-electro-mechanical behaviour,” in Hydrogel sensors and actuators. Editors Gerlach G. Arndt K.-F. 1 edn. (Berlin, Heidelberg: Springer-Verlag Berlin Heidelberg), 137–163. chap. 4.

[B143] Wallmersperger T. Ballhause D. (2008). Coupled chemo-electro-mechanical finite element simulation of hydrogels: II. Electrical stimulation. Smart Mater. Struct. 17, 045012–10. 10.1117/12.774639

[B144] Wallmersperger T. Leichsenring P. (2016). “Polymer gels as EAPs: models,” in Electromechanically active polymers: a concise reference. Editor Carpi F. (Cham: Springer), 277–278. chap. 3. 10.1002/pi.2790

[B145] Wallmersperger T. Kröplin B. Holdenried J. Gülch R. W. (2001). “A coupled multi-field-formulation for ionic polymer gels in electric fields,” Proceedings of SPIE, Bellingham, Washington, March 4-8 2001. 264–275. 10.1117/12.432655

[B146] Wallmersperger T. Kröplin B. Gülch R. W. (2002). “Numerical simulation of a coupled chemo-electric-formulation for ionic polymer gels in electric fields,” Proceedings of SPIE, Bellingham, Washington, March 17-21 2002. 4695, 303–314. 10.1117/12.475177

[B147] Wallmersperger T. Kröplin B. Gülch R. W. (2004a). Coupled chemo-electro-mechanical formulation for ionic polymer gels - numerical and experimental investigations. Mech. Mater. 36, 411–420. 10.1016/s0167-6636(03)00068-1

[B148] Wallmersperger T. Kröplin B. Gülch R. W. (2004b). “Modeling and analysis of chemistry and electromechanics,” in Electroactive polymer (EAP) actuators as artificial muscles: reality, potential, and challenges. Editor Bar-Cohen Y. 2 edn. (Bellingham, Washington: SPIE Press), 335–362. chap. 11.

[B149] Wallmersperger T. Kröplin B. Gülch R. W. (2004c). “Polyelectrolyte gels - basics, modelling and simulation,” in Chemo-mechanical couplings in porous media geomechanics and biomechanics. Editors Loret B. Huyghe J. M. 1 edn. (Vienna: Springer), 333–382.

[B150] Wallmersperger T. Ballhause D. Kröplin B. Günther M. Shi Z. Gerlach G. (2008). Coupled chemo-electro-mechanical simulation of polyelectrolyte gels as actuators and sensors. Electroact. Polym. Actuators Devices (EAPAD) 2008 (SPIE) 6927, 293–302.

[B151] Wallmersperger T. Ballhause D. Kröplin B. Günther M. Gerlach G. (2009). Coupled multi-field formulation in space and time for the simulation of intelligent hydrogels. J. Intelligent Material Syst. Struct. 20, 1483–1492. 10.1177/1045389X09105236

[B152] Wallmersperger T. Attaran A. Keller K. Brummund J. Günther M. Gerlach G. (2013a). “Modeling and simulation of hydrogels for the application as bending actuators. In *Intelligent Hydrogels* ,” in Progress in colloid and polymer science. Editor Gabriele Sadowski W. R. 1 edn. (Cham: Springer), 189–204.

[B153] Wallmersperger T. Keller K. Attaran A. (2013b). Polyelectrolyte gels as bending actuators: modeling and numerical simulation. Proceedings of SPIE, Bellingham, Washington, March 10-14 2013. 8687, 86872X. 10.1117/12.2009662

[B154] Warren M. R. Bajpayee A. G. (2022). Modeling electrostatic charge shielding induced by cationic drug carriers in articular cartilage using donnan osmotic theory. Bioelectricity 4, 248–258. 10.1089/bioe.2021.0026 36644714 PMC9811830

[B155] Woo S.-Y. Lubock P. Gomez M. Jemmott G. Kuei S. Akeson W. (1979). Large deformation nonhomogeneous and directional properties of articular cartilage in uniaxial tension. J. biomechanics 12, 437–446. 10.1016/0021-9290(79)90028-9 457697

[B156] Xu W. Zhu J. Hu J. Xiao L. (2022). Engineering the biomechanical microenvironment of chondrocytes towards articular cartilage tissue engineering. Life Sci. 309, 121043. 10.1016/j.heliyon.2024.e38112 36206835

[B157] Yang Y. Zhao Y. Engberts J. B. (2000). Stimuli response of polysoap hydrogels in aqueous solution and DC electric fields. Colloids Surfaces A Physicochem. Eng. Aspects 169, 85–94. 10.1016/S0927-7757(00)00420-9

[B158] Yew Y. K. (2006). Model development for numerical simulaiton of the behaviors of pH-stimulus responsive hydrogels. Ph.D. thesis. Singapore: National University of Singapore.

[B159] Yew Y. K. Ng T. Y. Li H. Lam K. Y. (2007). Analysis of pH and electrically controlled swelling of hydrogel-based micro-sensors/actuators. Biomed. Microdevices 9, 487–499. 10.1007/s10544-007-9056-4 17520372

[B160] Yuan Z. Li H. (2013). Modeling development and numerical simulation of transient nonlinear behaviors of electric sensitive hydrogel membrane under an external electric field. J. Biochips Tissue Chips 03. 10.4172/2153-0777.1000103

[B161] Yuan Z. Yin L. Jiang H. (2007). Numerical simulation of transient nonlinear behaviors of electric-sensitive hydrogel membrane under an external electric field. Microfluid. BioMEMS, Med. Microsystems V (SPIE) 6465, 282–288. 10.1117/12.697085

[B162] Zhang L. Miramini S. Smith D. W. Gardiner B. S. Grodzinsky A. J. (2015). Time evolution of deformation in a human cartilage under cyclic loading. Ann. Biomed. Eng. 43, 1166–1177. 10.1007/s10439-014-1164-8 25331101

[B163] Zhang X. Wang S. Chen X. Cui Z. Li X. Zhou Y. (2024). Bioinspired flexible kevlar/hydrogel composites with antipuncture and strain-sensing properties for personal protective equipment. ACS Appl. Mater. and Interfaces 16, 45473–45486. 10.1021/acsami.4c08659 39148460

[B164] Zhao S. Mehta A. S. Zhao M. (2020). Biomedical applications of electrical stimulation. Cell. Mol. Life Sci. 77, 2681–2699. 10.1007/s00018-019-03446-1 31974658 PMC7954539

[B165] Zhou X. Hon Y. C. Sun S. Mak A. F. T. (2002). Numerical simulation of the steady-state deformation of a smart hydrogel under an external electric field. Smart Mater. Struct. 11, 459–467. 10.1088/0964-1726/11/3/316

[B166] Zhou Z. Zheng J. Meng X. Wang F. (2023). Effects of electrical stimulation on articular cartilage regeneration with a focus on piezoelectric biomaterials for articular cartilage tissue repair and engineering. Int. J. Mol. Sci. 24, 1836. 10.3390/ijms24031836 36768157 PMC9915254

[B167] Zimmermann J. Farooqi A. R. van Rienen U. (2024). Electrical stimulation for cartilage tissue engineering - a critical review from an engineer’s perspective. Heliyon 10, e38112. 10.1016/j.heliyon.2024.e38112 39416819 PMC11481755

[B168] Zohdi T. I. (2017). Modeling and simulation of functionalized materials for additive manufacturing and 3d printing: continuous and discrete media: continuum and discrete element methods, 60. Cham: Springer.

[B169] Zou F. Xu J. Yuan L. Zhang Q. Jiang L. (2022). Recent progress on smart hydrogels for biomedicine and bioelectronics. Biosurface Biotribology 8, 212–224. 10.1049/bsb2.12046

[B170] Zuo X. Zhou Y. Hao K. Liu C. Yu R. Huang A. (2024). 3d printed all-natural hydrogels: flame-retardant materials toward attaining green sustainability. Adv. Sci. 11, 2306360. 10.1002/advs.202306360 PMC10797461 38098258

